# Dual-Polarization Bandwidth-Bridged Bandpass Sampling Fourier Transform Spectrometer from Visible to Near-Infrared on a Silicon Nitride Platform

**DOI:** 10.1021/acsphotonics.2c00451

**Published:** 2022-07-21

**Authors:** Kyoung Min Yoo, Ray T. Chen

**Affiliations:** † Department of Electrical and Computer Engineering, The University of Texas at Austin, 10100 Burnet Road, Austin, Texas 78758, United States; ‡ Omega Optics Inc., 8500 Shoal Creek Boulevard, Building 4, Suite 200, Austin, Texas 78757, United States

**Keywords:** spectrometer, Fourier transform spectrometer, bandpass sampling, near infrared, silicon nitride waveguide, tissue transparency window

## Abstract

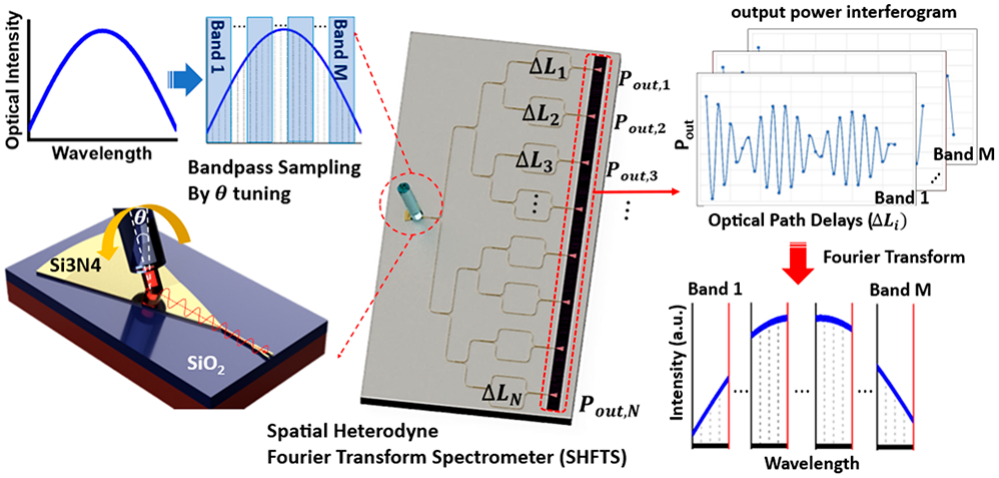

On-chip broadband optical spectrometers that cover the entire tissue transparency window (λ = 650–1050 nm) with high resolution are highly demanded for miniaturized biosensing and bioimaging applications. The standard spatial heterodyne Fourier transform spectrometer (SHFTS) requires a large number of Mach–Zehnder interferometer (MZI) arrays to obtain a broad spectral bandwidth while maintaining high resolution. Here, we propose a novel type of SHFTS integrated with a subwavelength grating coupler (SWGC) for the dual-polarization bandpass sampling on the Si_3_N_4_ platform to solve the intrinsic trade-off limitation between the bandwidth and resolution of the SHFTS without having an outrageous number of MZI arrays or adding additional active photonic components. By applying the bandpass sampling theorem, the continuous broadband input spectrum is divided into multiple narrow-band channels through tuning the phase-matching condition of the SWGC with different polarization and coupling angles. Thereby, it is able to reconstruct each band separately far beyond the Nyquist criterion without aliasing error or degrading the resolution. We experimentally demonstrated the broadband spectrum retrieval results with the overall bandwidth coverage of 400 nm, bridging the wavelengths from 650 to 1050 nm, with a resolution of 2–5 nm. The bandpass sampling SHFTS is designed to have 32 linearly unbalanced MZIs with the maximum optical path length difference of 93 μm within an overall footprint size of 4.7 mm × 0.65 mm, and the coupling angles of SWGC are varied from 0° to 32° to cover the entire tissue transparency window.

## Introduction

Owing to unique “fingerprint” signatures of molecular absorption and Raman spectra of the individual molecules, the optical spectrometer has been known as an indispensable tool to analyze the optical spectrum for a wide range of applications including biological and chemical analysis, medical diagnosis, and environmental and planetary monitoring, to name a few. Particularly, the near-infrared wavelength is beneficial for adoption in mammalian tissues because of its low absorbance, autofluorescence, and lower light-scattering loss from various mammalian biocells including hemoglobin, melanin, fat, lipids, water, and others, when compared to the shorter wavelengths.^1−3^ Also, a noninvasive in vivo diffuse optical characterization of human tissues using optical spectroscopy to assess mean absorption and reduced scattering spectra in an NIR tissue transparency window (∼650–1050 nm) opens a new possibility of monitoring various vital signatures of bioanalytes.^2,4,5^ Conventional spectrometers consist of movable mirrors, and free-space optic components are typically bulky and expensive. Moreover, they require sensitive and precise beam alignments, which place constraints on their applications in particular platforms accompanying environmental fluctuations, such as airborne or hand-held devices. However, the development of on-chip spectrometers based on photonic integrated circuits (PICs) has shown great promise in the sense that they can offer low-cost, portable, and robust spectroscopy, along with low power consumption and high reliability.^6^ In recent decades, a myriad of on-chip spectrometer devices have been demonstrated based on different operating schemes, such as dispersive optics using arrayed waveguide gratings (AWGs),^7−10^ echelle grating,^11^ metasurface elements,^12^ arrayed narrow-band filters,^13^ computational spectral reconstruction-based systems,^14^ and Fourier transform spectroscopy (FTS). Dispersive spectrometers, often called grating spectrometers or scanning spectroscopy, splits the wavelengths of input light into separate spectral ranges and collects each wavelength individually. A lot of grating-based devices have been studied and reported with sub-nanometer resolution in the visible (VIS) to near-infrared (NIR) range,^7−11,37,38^ but the gratings or slits on a dispersive device limit the amount of energy reaching the detector and the scan speed of spectroscopy because the individual wavelengths across the bandwidth have to be measured separately. Meanwhile, FTS is a technique that measures the spectrum with the interference of light instead of dispersion, so it does not separate energy into individual wavelengths for measuring the spectrum, offering advantages including high optical throughput and a multiplexing advantage, and, in turn, a larger signal-to-noise ratio (SNR) and faster data collection speed compared to the grating-based dispersive counterparts. Several on-chip FTS operation schemes were proposed, including stationary-wave integrated Fourier transform (SWIFT)^15,16^ spectrometers, microring resonator (MRR)-assisted Fourier transform (RAFT)^17^ spectrometers, and spatial heterodyne FTSs (SHFTSs).^18−21^ In the SWIFT scheme, the interference patterns created from the interference of two adjacent propagating waves through parallel waveguides are diffracted out-of-plane and monitored by the external detectors such as an array of photodetectors (PDs).^15^ To achieve the full interferogram completely, the pitch size of the PD arrays should be smaller than that of the interference pattern to avoid subsampling errors.^15^ Consequently, the resolution and bandwidth of the SWIFT spectrometer are highly limited by the minimum pitch size of the PD array, which puts a significant constraint on practical applications. To overcome this Nyquist–Shannon criterion, recent research reported a broadband SWIFT spectrometer integrated with a lithium niobate (LN) electro-optics modulator to retrieve the fully sampled interferogram without any moving components.^22^

The SHFTS consists of an array of unbalanced Mach–Zehnder interferometers (MZIs) with linearly increasing optical path delays (OPDs).^18,19^ In this concept, the output powers of each MZI configure each point of the spatial interferogram, which can be captured independently by a linear on-chip PD array, allowing the acquisition of the entire interferogram in a single capture without any moving parts or external analyzers. However, the spectral resolution and bandwidth of SHFTS are closely related to the number of MZIs and maximum OPDs, which always requires a balance to meet the limited chip-scale and detecting conditions. In other words, in a standard SHFTS configuration, there is a trade-off between the resolution and bandwidth; hence, achieving a fine resolution with broadband operation requires an unrealistically large number of MZI arrays, which increases the size of the device footprint significantly. For example, we previously demonstrated a standard SHFTS device with 24 MZI array for a resolution of 5 nm with a bandwidth of 60 nm centered at λ_o_ = 900 nm in the silicon nitride (Si_3_N_4_) platform.^23^ However, to cover the VIS to NIR tissue transparency window, it requires broadband operation with a ∼400 nm bandwidth. To increase the bandwidth while maintaining a 5 nm resolution, it requires a more than 160 MZI array, which is impractical to be implemented as an on-chip device in that the amount of energy reaching each MZI will be significantly limited after dividing it into 160 arrays as well as a large device footprint. To overcome this restriction, several approaches were proposed such as thermally tuned MZI structures,^24,25^ RAFT,^17^ and applying the compressive-sensing scheme.^26^ Especially, RAFT consists of a thermally tunable MZI cascaded with a thermally tunable MRR and demonstrated spectrum retrieval results from one output channel with a resolution of 0.47 nm, bypassing the Rayleigh criterion of the standard MZI-based FTS device by implementing a high-resolution MRR filter.^17^ In spite of the advantages of the thermo-optic effect contributing to the small-footprint and high-resolution performance, it takes a longer time to collect each point of the thermally induced interferogram separately with corresponding incremental changes of temperature (Δ*T*_step_), controlled by the thermoelectric temperature controller (TEC) to achieve a uniform sampling of the thermo-optical-induced OPD, which also makes the overall system configuration complex compared to the standard SHFTS.

In this paper, instead of adding additional active photonic components, we propose and experimentally demonstrate an alternative approach using a bandpass sampling theorem with standard SHFTS configuration integrated with a subwavelength grating coupler (SWGC) to achieve the overall bandwidth covering the entire NIR tissue transparency window (650 to 1050 nm) with <5 nm resolution. To build the on-chip SHFTS device for NIR wavelength, we chose the Si_3_N_4_ platform due to its low material absorption loss over the wide spectral range from 400 nm up to 2.3 μm as well as the low phase noise and error due to the lower refractive index contrast between the silicon dioxide (SiO_2_) cladding (*n*_SiO_2__ ≈ 1.46 @ λ = 900 nm) and the Si_3_N_4_ core (*n*_Si_3_N_4__ ≈ 2.01 @ λ = 900 nm)^27^ compared to the silicon waveguide.^28^ Also, the refractive index of Si_3_N_4_ varies depending on the deposition techniques and the quality of films. Typically, low-pressure chemical vapor deposition (LPCVD)-deposited Si_3_N_4_ has a higher refractive index than the plasma-enhanced CVD (PECVD)-deposited Si_3_N_4_, so we decided to use LPCVD-deposited Si_3_N_4_ and applied corresponding refractive index values for the device design and simulation.^28^ Finally, we have developed and fabricated the first prototype SHFTS chip on a CMOS-compatible Si_3_N_4_ platform and demonstrated experimental results.

## Results

### Concept and Principle

The theory and principle of the standard SHFTS have been demonstrated by Florjańczyk et al.^18^ previously. As briefly mentioned in the Introduction, SHFTS consists of an array of unbalanced MZIs with linearly increasing OPDs with a constant increment across the array configuring the spatial interferogram. For a given single-input source, the phase change from each MZI is converted into an intensity change based on interferometric schemes. The input spectrum can be retrieved through the discrete Fourier transform (DFT), which can be written as^18^
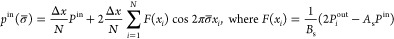
1Here, *P*^in^ is the input power, *N* is the number of MZIs, σ is the wavenumber, and σ̅ = σ – σ_min_ is the shifted wavenumber, where *σ*_min_ represents the minimum wavenumber at the Littrow condition; at the Littrow condition, the phase delays in different MZIs are integer multiples of 2π, so the output powers of each MZI (*P*_*i*_^out^) are constant. Δ*x* is the maximum interferometric delay, that is Δ*x* = *n*_eff_Δ*L*_max_, where *n*_eff_ is the effective index of the strip waveguide and Δ*L*_max_ is the maximum path delay of the most unbalanced MZI. The spatial interferogram *F*(*x*_*i*_) is discretized at *N* equally spaced OPD values *x*_*i*_ (0 ≤ *x*_*i*_ ≤ Δ*x*), which is defined as *x*_*i*_ = *n*_eff_Δ*L*_*i*_, where Δ*L*_*i*_ is the path length difference of the *i*th unbalanced MZI. The input power *P*^in^ is constant for all the MZIs, and *P*_*i*_^out^ represents the output power of the *i*th MZI with the coupling and loss coefficients of the MZI components *A*_s_ and *B*_s_. As the wavenumber of monochromatic input σ changes from the Littrow wavenumber, the *P*_*i*_^out^ distribution becomes periodic, and different wavenumbers create different periodic patterns. Subsequently, a polychromatic input signal, which can be considered as a superposition of monochromatic constituents, creates a corresponding spatial interferogram pattern formed by a superposition of the respective periodic *P*_*i*_^out^ fringes from monochromatic input. The resolution of spectrometers, represented by the wavenumber resolution δσ, is determined by the maximum interferometric delay Δ*x*. To resolve two monochromatic signals separated by δσ at the most unbalanced MZI with an OPD of δσ, the phase change from respective interferograms of σ and σ + δσ should differ by one fringe (2π); that is Δφ = 2π(σ + δσ)Δ*x* – 2πσΔ*x* = 2π; in turn,^18^
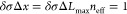
2Thus, the maximum path delay of the MZI array (Δ*L*_max_) can be designated as follows:
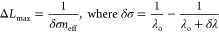
3where λ_o_ is the center wavelength and δλ is the wavelength resolution. The number of MZIs in the array (*N*) is equivalent to the discrete sampling points in the spatial interferogram. Based on the Nyquist–Shannon sampling theorem, the minimum sampling points (*N*_min_) required to fully reconstruct the input spectrum (*p*_in_(σ̅)) within the band-limit range is determined as follows:
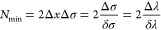
4where Δσ and Δλ are the wavenumber and wavelength bandwidth of the spectrometer. Based on the sampling theory, the wavenumbers that are equally distributed above and below the Littrow wavenumbers produce the same interference fringe patterns, and the input spectrum reconstructed by the DFT of the spatial interferogram creates the wavenumber-shifted replicas of the original transform *p*^in^(σ̅) above and below the band limit as shown in Figure S1a. Consequently, when the input spectrum contains the signals outside of the band limit exceeding the bandwidth, the reconstruction aliasing error due to the overlap of these copies (flipped images) makes the retrieved spectrum indistinguishable, as described in Figure S1b, and this intrinsic constraint puts a limitation on retrieving the broadband continuum input signals from the Fourier transform system.

In signal processing, a technique that is known as bandpass sampling or undersampling has been used to reconstruct the signal with a sampling rate below the minimum Nyquist rate (eq 4) using a bandpass filter.^29,30^ In this paper, we exploit this concept in the SHFTS configuration to solve the trade-off condition between the bandwidth and resolution by employing the bandpass filter to divide the broadband-continuous spectrum into multiple narrow-band channels. Thereby, it is able to reconstruct each band without aliasing error, rather than retrieving the signals all together in a single band. As a consequence, we were able to achieve the broad overall bandwidth coverage (Δλ_overall_) without degrading the resolution.

The grating coupler (GC) is one of the essential components to build PIC chips for the fiber-to-chip coupling.^31,32^ In general, the narrow-bandwidth operation, polarization (transverse-electric (TE) and transverse-magnetic (TM)), and angular dependencies of GC are considered as intrinsic disadvantages of employing the broadband coupler. However, these properties are very beneficial for the bandpass-sampling scheme, since it can be implemented as a coupler as well as a polarization-selective bandpass filter; in addition, the coupling wavelength can be shifted by the coupling-angle (θ) tuning based on the phase-matching condition,^33^ which makes it a tunable-bandpass filter.

**Figure 1:**
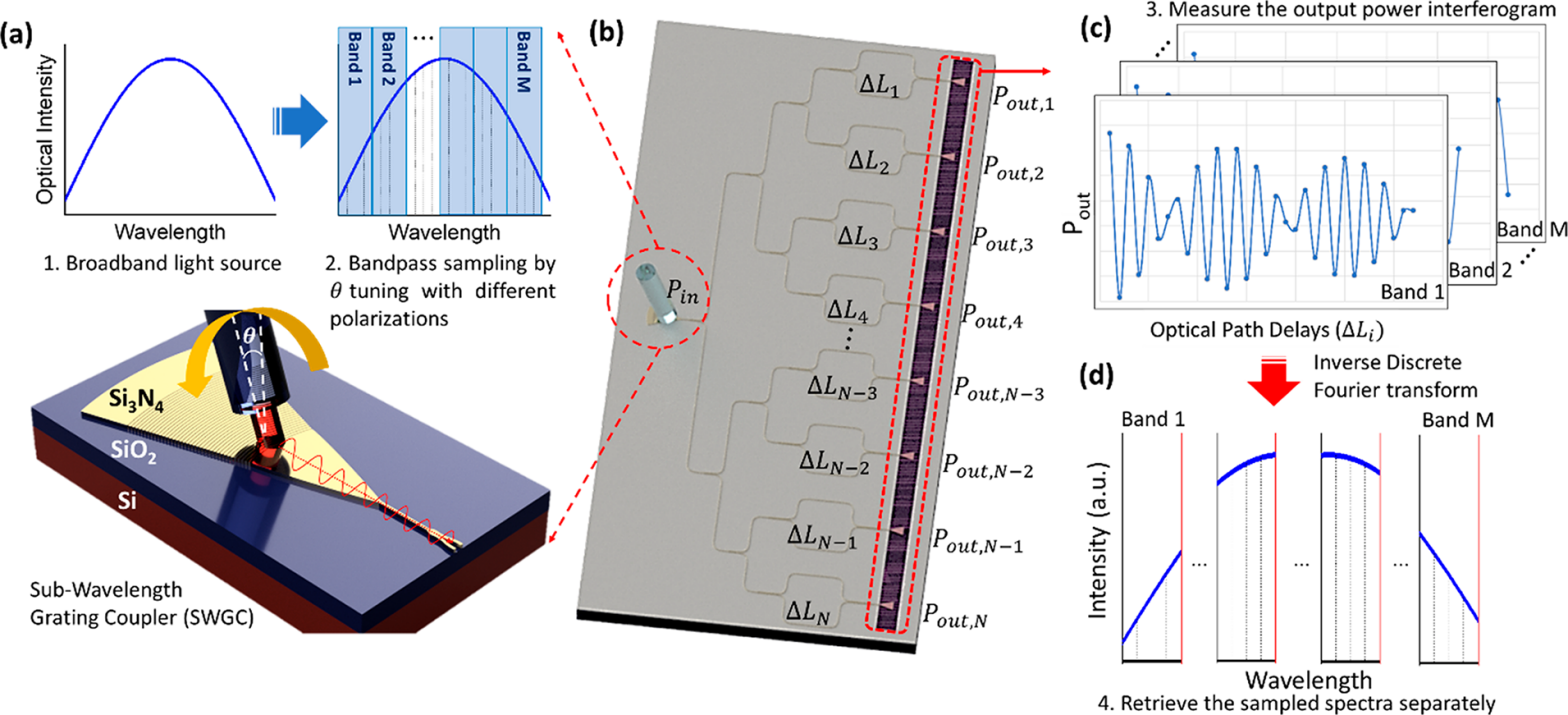
Schematic illustration of the operation principle of a bandpass sampling SHFTS integrated with an SWGC. (a) Schematic illustration of the SWGC operation; the SWGC serves not only as a coupler but also as a tunable-bandpass filter to divide the broadband spectrum into multiple narrow-band channels by changing the coupling angle with different polarization. (b) Si_3_N_4_ SHFTS chip with an array of N-MZIs with linearly increasing OPD; the broadband spectrum is divided into M-spectra based on the Nyquist rate and can be reconstructed separately. (c) The spatial interferograms of each discrete narrow band consist of output powers from the MZI array; phase change from each MZI is converted into the intensity change. (d) The sampled narrow-band spectra can be reconstructed by Fourier transform.

Figure 1 demonstrates the overall design and operation principle of a Si_3_N_4_ bandpass sampling SHFTS integrated with an SWGC that covers the entire NIR tissue transparency window (650–1050 nm). The SHFTS consists of an array of MZIs with a single SWGC input port (Figure 1b). The broadband input signal is coupled from the fiber to the waveguide through the SWGC (Figure 1a), and the input spectrum is bandpass-filtered based on the SWGC’s phase-matching condition, which can be tuned by the coupling angle (θ) with different polarizations. Namely, the broadband input spectrum is bandpass sampled into multiple narrow bands by different phase-matching conditions, which completely divide the coupling wavelengths into *M* number of discrete narrow-band channels of the SHFTS. The coupled light is equally divided into linearly unbalanced MZIs through the cascaded splitters, and the intensities of each MZI output powers are measured, forming a spatial interferogram for each sampled band (Figure 1c). Then, each sampled input spectrum can be fully recovered using eq 1 without overlapped aliases (antialiasing) (Figure 1d).

To build the on-chip SHFTS with low-loss operation in the NIR tissue transparency window (∼650–1050 nm), we designed and optimized the Si_3_N_4_ passive components including the strip waveguide, multimode interferometer (MMI) splitter and combiner, and the SWGC using the Lumerical simulation tool. In the design of the prototype, we used the SWGCs for the MZI output ports to measure the output powers, which can be further integrated with a PD array by virtue of the modern PIC technology,^34^ providing much more compact and stable devices. In the following sections, we will demonstrate the simulation and experimental results of the optimized passive components and the first prototype of the SHFTS device. The optimal design and simulation results of the Si_3_N_4_ strip waveguide (Figure S2) and MMI splitter/combiner (Figure S5) are described in the Supporting Information.

### Subwavelength Grating Coupler

A conventional GC with shallow etched gratings requires additional alignment lithography steps, which make the whole fabrication process complex. To alleviate the fabrication complexity, several through-etched GC designs using subwavelength grating (SWG) structures to reduce the Fresnel back reflection have been reported in various platforms.^35,36^ In this work, by implementing the SWG structures between the major Si_3_N_4_ gratings, we were able to get a through-etched structure that can be patterned and etched altogether with other passive components without additional alignment patterning steps.

**Figure 2:**
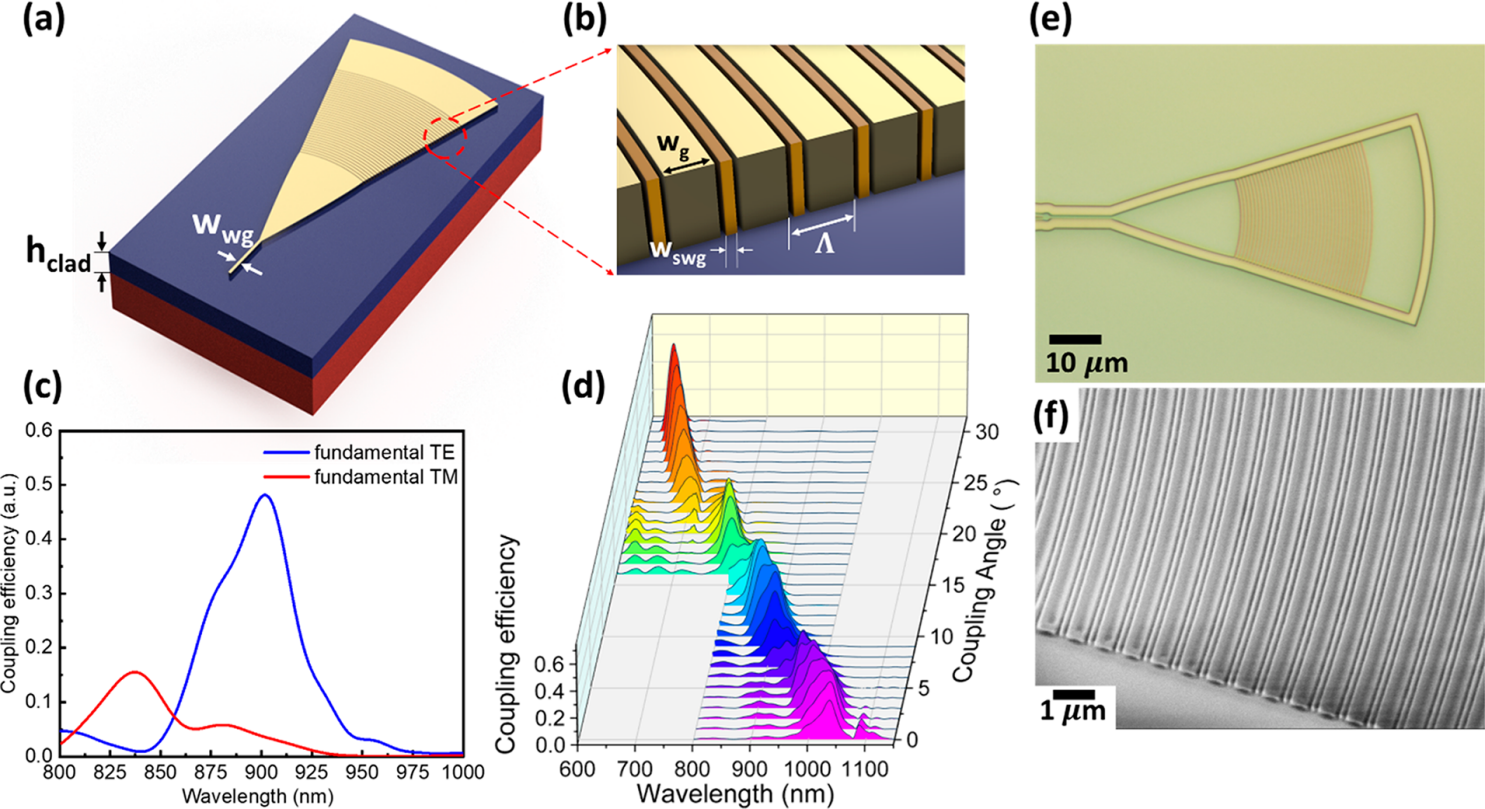
SWGC design and simulation results. (a) 3D schematic illustration of the Si_3_N_4_ SWGC and (b) zoomed-in image of the grating structures. (c) Coupling efficiencies (*S*-parameters) of the optimized SWGC with θ = 11° for the fundamental TE and TM modes as a function of the wavelength. (d) XYZ plot showing the simulation result of the SWGC TE mode coupling wavelength (λ_o_) shift by tuning the coupling angle from 0° to 31°; *x*-axis: wavelength, *y*-axis: coupling efficiency, *z*-axis: coupling angle. (e) Optical microscope and (f) SEM images of the fabricated SWGC.

Also, it is known that the central coupling wavelength λ_o_ of the grating coupler is determined as in the following equation based on the phase-matching condition.^33^
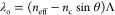
5where *n*_eff_ is the effective index of the grating, *n*_c_ is the refractive index of the cladding, θ is the coupling angle, and Λ is the grating period. Therefore, the coupling wavelength λ_o_ can be shifted by θ tuning based on eq 5. Here, we designed the Si_3_N_4_ SWGC integrated with an SHFTS chip, serving not only as a coupler but also as a tunable bandpass filter for the bandpass sampling of the broadband input signals. The schematic illustration of the SWGC is shown in Figure 2a and b.

A simulation model (Figure S3a) has been created using 2D and 3D finite-difference time-domain (FDTD) simulation to optimize the device for the fundamental TE mode with small insertion loss as well as small back reflection to the waveguide. Design parameters including the grating period (Λ), the width of the grating (*w*_g_), and the width of the subwavelength grating (*w*_swg_) were optimized with the built-in particle swarm algorithm in the simulation tool. Furthermore, the thickness of the SiO_2_ bottom cladding was optimized as *h*_core_ = 2.8 μm to get the maximum coupling efficiency as shown in Figure S3c. The refractive index profile and *E*-field of the optimized SWGC structure are shown in Figure S3b and d, showing that the light is coupled from the fiber to the waveguide through the SWG structures. Figure 2c shows the coupling efficiency of the optimized SWGC with θ = 11° for both TE and TM modes as a function of the wavelength, extracted from the *S*-parameters of the input and output ports. The coupling efficiency unit is normalized as a maximum of 1, indicating that 100% of light is coupled from the fiber to the waveguide, and the reciprocal *S*-parameters indicated that the coupling efficiency from the fiber to the waveguide is identical with the opposite direction. Moreover, we can see the coupling selectivity between the TE mode and TM mode, and the SWGC has a ∼3 dB coupling loss with a TE mode at λ_o_ = 900 nm, along with the 3 dB bandwidth of ∼50 nm. Finally, we swept the coupling angle from θ = 0° to 31° and monitored the λ_o_ shifts from 1030 to 650 nm, and the results in the XYZ plot and XY plot are shown in Figure 2d and Figure S4. The maximum coupling efficiency of ∼65% was achieved at λ_o_ = 650 nm with θ = 29°, and the center wavelength shifts to a longer wavelength up to 1030 nm along with the 3 dB bandwidth increases from ∼30 nm to 70 nm as the coupling angle decreases to surface normal. On the basis of the optimal design and simulation results, we fabricated the device and inspected the dimension deviation by scanning electron microscope (SEM) due to the fabrication error as in Table S2, and the corresponding optical microscope and SEM images can be found in Figure 2e,f and Figure S8.

Since these deviations of the device dimensions are critical to the phase-matching condition of SWGC and in turn the coupling efficiencies and wavelengths, we carefully examined the simulation results with the real-fabricated dimensions that are shown in Figure S3e. Comparing with the ideal simulation results, we observed a slight red-shift of the overall spectrum. Moreover, we found that the SWGC cannot effectively couple some of the wavelength ranges with the TE mode, especially between the λ ≅ 700 and 800 nm. We presumed that this is due to the vertical destructive interference between the Si_3_N_4_ and SiO_2_ layers. To address this issue and couple the entire wavelength range, we utilized the TM mode, which has a different phase-matching condition from the TE mode. Figure S3f shows the TM mode simulation results using the same SWGC structures. Although the SWGC structure is optimized for the TE mode, the SWGC can couple the TM mode even better than the TE mode especially for the shorter wavelengths, which allows bridging the TE mode-forbidden wavelengths between 700 and 800 nm. Accordingly, the entire wavelength from 650 to 1050 nm can be bandpass filtered and coupled into the SHFTS with corresponding polarization and coupling angle (θ) conditions.

### Bandpass Sampling SHFTS Design

**Figure 3:**
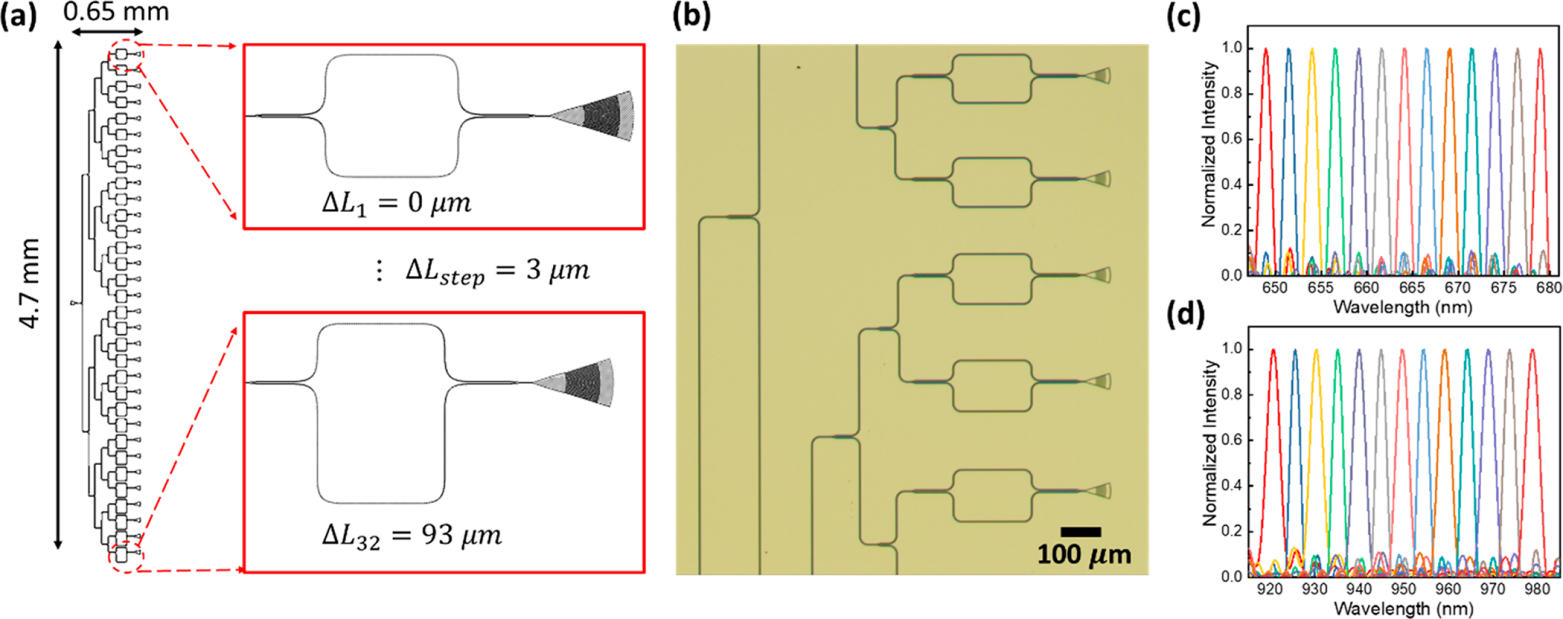
SHFTS comprising 32 MZI arrays integrated with SWGC inputs and outputs. (a) Final outlook of the SHFTS device; the overall size is around 4.7 mm × 0.65 mm. (b) Optical microscope image of the fabricated device. (c) Simulation results of the SHFTS; reconstructed spectrum in band 1 and (d) band 7 with minimum resolvable wavelength detuning.

Using the optimized Si_3_N_4_ components described above, we designed and analyzed the bandpass sampling SHFTS. Based on eqs 3 and 4, the number of MZI arrays (*N*) and the maximum OPDs (Δ*L*_max_) are designed to specify the bandwidth (Δλ) and the resolution (δλ) of the SHFTS. First, the bandwidth of the SHFTS is designed to be wider than the 3 dB bandwidths of the SWGC, preventing the overlap of sampled aliases. In other words, the bandwidths of each narrow-band channels of SHFTS must be able to fully cover the coupled wavelengths from the SWGC. Here, we designed the SHFTS with *N* = 32 and Δ*L*_max_ = 93 μm (Figure 3a) to have δλ < 5 nm and Δλ = 30–80 nm at the wavelength range of λ = 650–1050 nm. The total wavelength window (650–1050 nm) is divided into eight discrete channels based on the Nyquist rate and can be reconstructed separately. The optical microscope image of the fabricated device in Figure 3b shows part of an MZI array. In order to better examine the resolution and bandwidth of the SHFTS, we built the interconnect simulation model of the SHFTS using the optimized components, and the λ_o_ of single peak input signals were swept from 647 to 1060 nm and the signals were reconstructed from the SHFTS to verify the minimum resolvable wavelength detuning in each channel. The spatial interferograms of the output power from 32 MZIs were measured by optical power meter, and the input signals were retrieved by using eq 1 in MATLAB. The overall configurations of the narrow-band channels including the corresponding bandwidths and resolutions are shown in Table 1, and the reconstructed spectra with minimum resolvable wavelength detuning from bands 1 and 7 are shown in Figure 3c and d, respectively.

**Table 1:** Discrete Narrow-Band Channels of a Bandpass Sampling SHFTS

band	start wavelength [nm]	end wavelength [nm]	bandwidth [nm]	resolution [nm]
1	647	680	33	2.06
2	680	715	35	2.19
3	715	755	40	2.50
4	755	800	45	2.81
5	800	855	55	3.44
6	855	915	60	3.75
7	915	985	70	4.38
8	985	1060	75	4.69

### Experimental Results

By virtue of a through-etched SWGC design, we were able to pattern and etch the whole device in one lithography and etching step without any additional alignment lithography process. The final footprints of the fabricated chip including the loss characterization patterns and bandpass sampling SHFTS device are shown in Figure S7.

Then, we built the measurement setup to test the fabricated device. The schematic diagram and real picture of the measurement setup are shown in Figure S9. We used the broadband supercontinuum light source (NKT Photonics SuperK laser) to examine the performances of the fabricated SWGC and bandpass sampling SHFTS.

First of all, to verify that the broadband light is coupled to the PM-SMF successfully, we measured the fiber-coupled SuperK laser’s spectrum by the optical spectrum analyzer (OSA) directly. Figure S10 shows the pictures of the SuperK laser coupling setup and the OSA measured optical spectrum from the fiber, and we examined that the broadband light is coupled to the PM-SMF successfully with the maximum total optical power of ∼130 mW. Using the measurement setup and the loss characterization devices consisting of different lengths of waveguides, the propagation losses of the Si_3_N_4_ strip waveguides were measured as ∼2.5 dB/cm by the cut-back method (Figure S11). The power loss from the MMI splitter is wavelength and polarization dependent, and the results are shown in Figure S5d and e.

**Figure 4:**
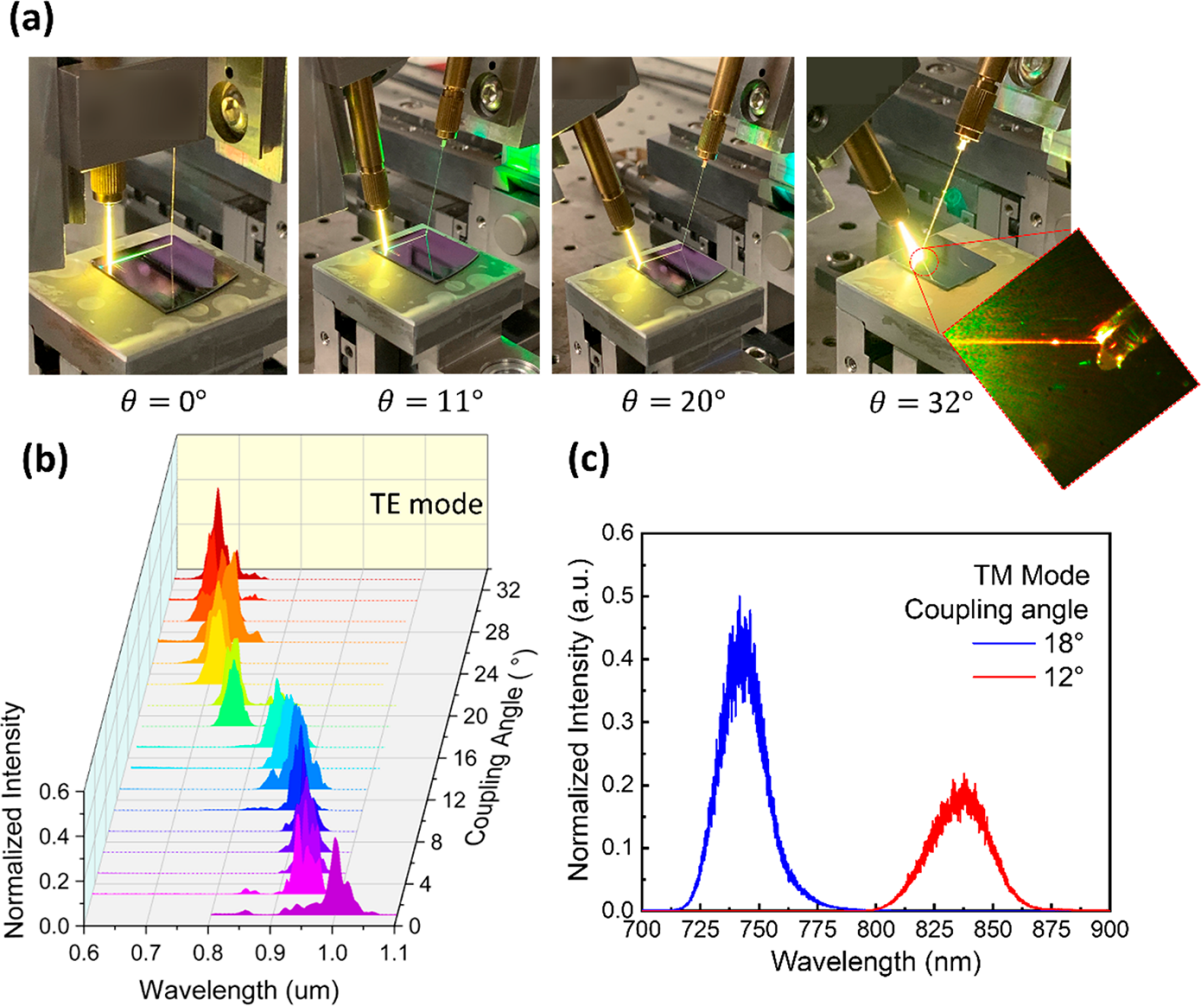
SWGC output measurement results. (a) Pictures of the fiber to SWGC coupling setup with different coupling angles; the enlarged picture shows the red-color guided light through the waveguide, coupled to the output fiber. (b) XYZ plot of the coupling efficiencies using the TE mode; *x*-axis: wavelength, *y*-axis: coupling angle, *z*-axis: normalized coupling efficiency. (c) XY plot of the coupling efficiencies using the TM mode; blue line: θ = 18°, red line: θ = 12°.

Then, the output spectra of the SWGC were measured with different coupling angles to test the coupling efficiencies and the wavelength shifting. The input and output coupling angles were equally controlled from 0° to 32°, as shown in the pictures in Figure 4a, and the output fiber was connected to the OSA to measure the spectrum directly.

Figure 4b and c show the SWGC spectrum measurement results by the OSA with TE and TM modes. With the fundamental TE mode (Figure 4b), the SWGC shows 4.5–8.5 dB losses, and the center wavelength λ_o_ shifts from 650 nm to 1000 nm with the corresponding 3 dB bandwidth changing from 30 nm to 70 nm as the coupling angle changes from 32° to 0°. More detailed coupling loss data based on the coupling angle are shown in Figure S12. The TM mode coupling efficiencies with the two different coupling angles θ = 18° and 12° were also measured for λ_o_ ≅ 740 and 835 nm, as shown in Figure 4c. On the basis of these results, we verified the coupling wavelength shifting range and efficiencies of the SWGC experimentally, covering the entire tissue transparency window (λ = 650–1050 nm). As a consequence, the SuperK broadband input signal can be bandpass filtered and divided into the multiple narrow-band channels shown in Table 1 through the SWGC and coupled into the waveguide.

**Figure 5:**
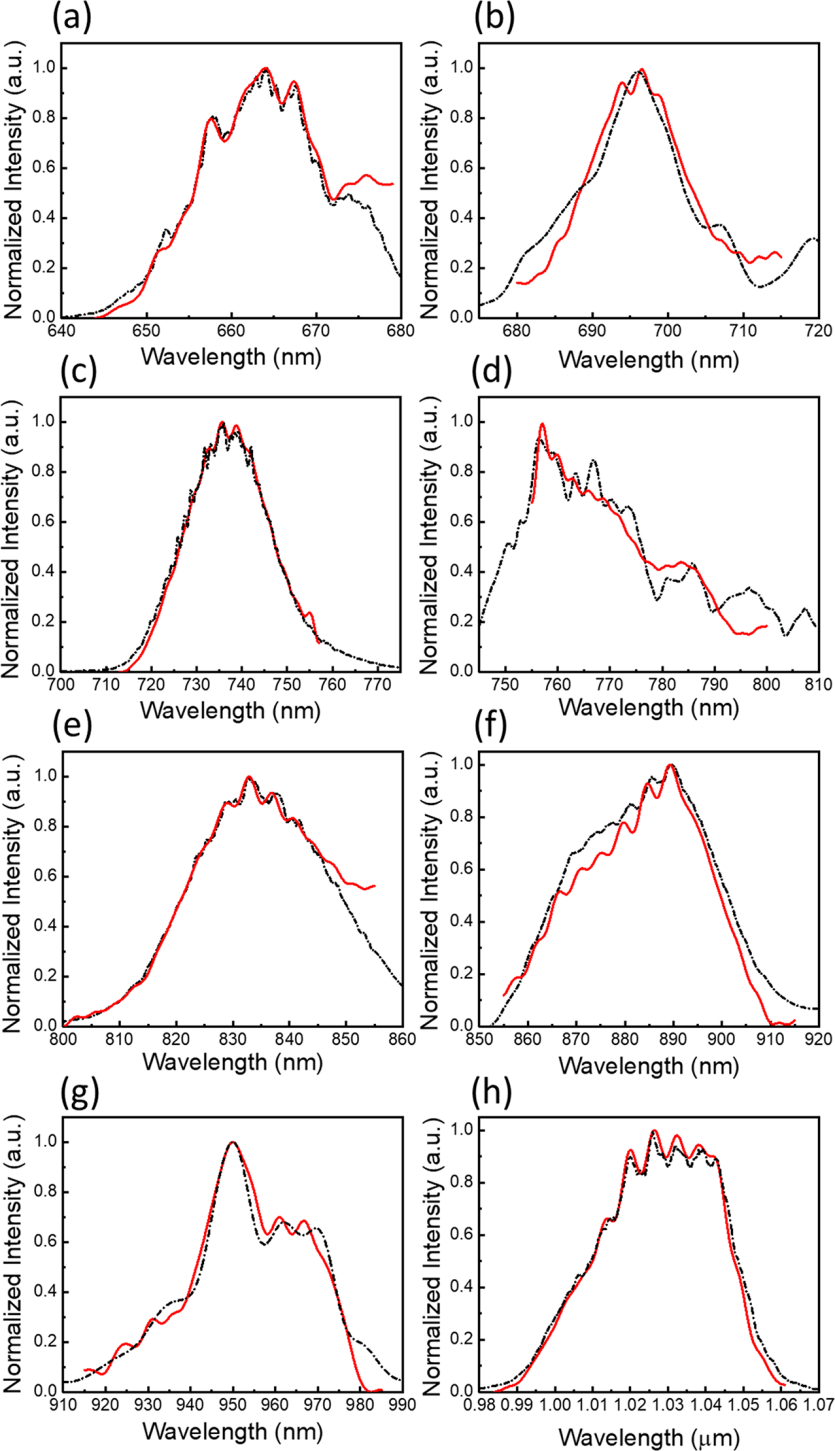
Retrieved spectra from the bandpass sampling SHFTS (red solid lines) and direct measured spectra from the OSA (black dotted lines) with different SWGC coupling conditions of (a) TE mode θ = 32°, (b) TE mode θ = 25°, (c) TM mode θ = 18°, (d) TE mode θ = 20°, (e) TM mode θ = 12°, (f) TE mode θ = 14°, (g) TE mode θ = 4°, and (h) TE mode θ = 0°.

Finally, using the same measurement setup, we coupled the SuperK laser light into the bandpass sampling SHFTS and measured the output powers of each MZI’s interferogram using different SWGC coupling angles to reconstruct the input spectrum. The single input signal is equally divided into 32 MZI channels by the cascaded MMI splitters, and the output powers of each MZI are measured separately by the optical power meter. The optical images of the light-coupled SHFTS chip can be found in Figure S13. We have measured the optical powers using eight different coupling conditions, which are θ = 32°, 25°, 20°, 14°, 4°, and 0° with TE polarization and θ = 18° and 12° with TM polarization to retrieve the entire tissue transparency wavelength range from 650 to 1050 nm. The experimental output power measurement results from 32 MZIs using different SWGC coupling conditions are shown in Figure S14. After we collected the output powers from each MZI, we reconstructed the optical spectrum of the bandpass-sampled signals by the MATLAB code based on the DFT equation (eq 1), and the results are shown in Figure 5. To validate the spectrum retrieval accuracy, the FTS retrieved results (red solid lines) were compared with the direct OSA measurement results (black dotted lines). The retrieved spectra are well matched with the direct OSA measurement results, but the discrepancies are mainly due to the optical phase errors induced from the etching surface and sidewall roughness and detecting noise from the optical power meter. Figure S15 shows the overall retrieved spectra from λ = 650–1050 nm by the superposition, and the intensity normalization is applied based on the coupling and loss values shown in Figures S5d,e and S12.

In conclusion, we experimentally retrieved the input signals from λ = 650 nm to 1050 nm with eight discrete narrow-band channels by the bandpass sampling SHFTS through SWGC coupling.

## Discussion

Our proof-of-concept experiment demonstrates the broadband spectrum retrieval performance using the broadband supercontinuum light source and bandpass sampling SHFTS, representing the overall spectrometer bandwidth coverage (Δλ_overall_) of 400 nm without compromising the spectrometer resolution. To the best of our knowledge, this is the first demonstration of the broadband FTS covering the entire tissue transparency window on a silicon nitride platform without adding additional active photonic components for electrical or thermal enhancements.

Previously reported miniaturized spectrometers at VIS–NIR wavelength ranges (Table 2) either were limited to the narrow bandwidth coverage or had poor resolution, or the measurement system requires a too large and complicated configuration,^11^ which cannot be implemented in hand-held or portable applications. In comparison to these spectrometers including thermally tunable FTS devices, the bandpass sampling FTS concept stands out in the sense that it provides broadband coverage while maintaining a fine resolution and relatively small size of the device footprint, without external active photonic components to introduce additional thermally or electrically induced OPDs. Moreover, the bandpass wavelength ranges are determined by the coupling angle of the SWGC, and each discrete channel can be retrieved in a single capture with a fixed coupling angle, which is beneficial to the real-time biosensing applications especially when we only need to scan a specific window.

**Table 2:** Comparison of Miniaturized Spectrometers at the VIS–NIR Wavelength Range (650–1050 nm)

schemes	principles	platforms	resolution [nm]	bandwidth [nm]	range [nm]	no. channel	footprint [mm^2^]
dispersive optics	waveguide grating diffraction^37^	SiON	40	800	300–1100		35
	MRR assisted AWG^7^	SiN	0.75	52.5	831–883	70	1.44
	AWG^8^	SiN	1.5	60	830–890	40	0.62
	AWG^9^	SiN	0.5	4	757–762	8	2.8
	AWG^10^	SiON	5.5	215	740–955	41	340
	planar echelle gratings and single photon detection^11^	SiN/NbN^a^	7	1400	600–2000	200	∼30
	folded metasurface^12^	SiO_2_/Si/Au	1.2	100	760–860		7
narrow-band filters	linear variable optical filter^13^	multilayered films (SiO_2_/TiO_2_)	2.2	170	570–740		100
reconstructive	PC slabs^14^	SOS	1	200	550–750	36	0.044
Fourier transform	SWIFT^15^	SiN	6	100	800–900		0.1
	bandpass sampling SHFTS (this work)	SiN	2–5	400	650–1050	32	3

a Cryogenic low-noise amplifiers are required for the measurement.

Despite the advantages of the bandpass sampling SHFTS described above, our prototype result leaves much room for further improvement for the monolithic integrated circuits and chances for biosensing applications, which require the integration of the on-chip light source and PD array. Specifically, the current prototype device’s scanning speed is significantly limited by the fiber alignment and angle tuning to cover the entire bandwidth. In other words, each single separate band can be retrieved in a single capture with a fixed coupling angle, but scanning eight separate bands requires the coupling angle tuning and alignment with the grating couple for each band, which can be a slow speed operation. Moreover, to exploit the on-chip integrated broadband light source into the bandpass sampling SHFTS scheme, a proper method that can substitute the SWGC and fiber angle tuning in this work should be devised as an on-chip tunable-bandpass filter. Several studies have reported on-chip bandpass filter structures using a single-channel optical bandpass filter based on plasmonic nanocavities,^39^ gratings,^40,41^ and cascaded ring resonators^42^ or MZI structures,^43^ which can be potentially integrated with the SHFTS configuration. In the Supporting Information, we discuss the possibilities and challenges of the integration with the on-chip broadband light source and PD array, which provide guidelines to further improve the performances toward the fully integrated portable spectrometers for lab-on-a-chip Raman or absorption spectroscopy systems.

## Conclusion

In this paper, we designed and experimentally demonstrated the bandpass sampling SHFTS integrated with an SWGC on the Si_3_N_4_ platform to achieve broad bandwidth coverage (Δλ_overall_ = 400 nm) without compromising the spectrometer resolution (δλ ≈ 2–5 nm) in the VIS–NIR tissue transparency window (Δλ = 650–1050 nm). Unlike the standard SHFTS operating scheme, the bandpass sampling theorem is applied by tuning the coupling angle of the SWGC, substituting the tunable-bandpass filter in our design. Namely, the SWGCs were used not only as a fiber to waveguide coupler but also as an antialiasing filter, dividing the continuous broadband spectrum into multiple narrow-band channels. Then, the original broadband spectrum can be reconstructed by the superposition of each retrieved narrow-band spectra. We optimized the low-loss passive components to build the MZI structures, including the strip waveguide, MMIs, and SWGC, and the SHFTS is designed to have 32 linearly unbalanced MZIs with the maximum OPD (Δ*L*_max_) of 93 μm, with a total footprint size of ∼4.7 mm × 0.65 mm. To the best of our knowledge, this is the first experimental demonstration of the broadband FTS covering the entire tissue transparency window on a silicon nitride platform without adding additional active photonic components.

## Methods

### Device Simulation and Optimization

The simulation software Lumerical MODE and FDTD were used to analyze and optimize the passive components. The internal optimization tool with the particle-swarm-algorithm was used to generate a number of structures with different dimensions in specific ranges and get the maximum coupling efficiency or minimum losses as needed. Also, the *S*-parameters from the input and output ports of SWGC and MMIs were calculated from the FDTD simulation to monitor the light transmission characteristics, and the *S*-parameters from the optimized passive components were used to build the SHFTS model in the interconnected photonic circuit simulation.

### Si_3_N_4_ SHFTS Device Fabrication

The Si_3_N_4_-on-SiO_2_ wafers were prepared with a 220 nm thick LPCVD-grown Si_3_N_4_ on a 2.8 μm thick SiO_2_ bottom cladding on a silicon substrate (Figure S6a) from Rogue Valley Microdevices Inc. Then, a ∼400 nm thick e-beam resist (ZEP-520A) is deposited on top of the Si_3_N_4_ layer by spin-coating. The patterning is done by a JEOL e-beam (JBX-6000FS) lithography tool, followed by developing in *n*-amyl acetate for 2 min and rinsing in isopropyl alcohol (IPA) (Figure S6b). Following this, the pattern is transferred to the Si_3_N_4_ layer by reactive-ion etching (RIE) (Figure S6c). Finally, the remaining resist and polymers are cleaned using removal PG followed by cycles of acetone/IPA postprocess treatment (Figure S6d).

### Measurement Setup

The unpolarized broadband supercontinuum light source (NKT Photonics SuperK Versa), which covers the wavelength range from 550 to 1750 nm, is coupled to the polarization-maintaining single-mode fiber (Thorlab PM780-HP, PM630-HP) using a Glan-Thompson linear polarizer (Newport 5525) and microscope objective lens (100×) for TE or TM mode transmission. The fibers are mounted on the goniometer stages to control the coupling angles for both input and output fibers. The device is placed on the flat stage, and the input/output fibers are aligned with the SWGCs to couple the light to the waveguide, monitored by the optical microscope. Finally, the output fiber is connected to the optical power meter (Newport 2936-C) to measure the output power.

## Supporting Information Available

Additional simulations, experiment details, and photographs including the description of the Si_3_N_4_ strip waveguide and MMI design and further discussion about the on-chip broadband light source and detector integration and biosensing applications (PDF)
