# Supplementary material for: Dual-Polarization Bandwidth-Bridged Bandpass Sampling Fourier Transform Spectrometer from Visible to Near-Infrared on a Silicon Nitride Platform
Source: ACS Photonics. 2022 Jul 21;9(8):2691–701. doi: 10.1021/acsphotonics.2c00451 (PMC9390785; doi:10.1021/acsphotonics.2c00451)
Supplement: Supplementary file 1 — ph2c00451_si_001.pdf [file ph2c00451_si_001.pdf]

Supporting Information

Dual-polarization bandwidth-bridged bandpass sampling Fourier transform spectrometer from visible to near-infrared on a silicon nitride platform

KYOUNG MIN YOO[1,3] AND RAY T CHEN[1,2,4]

[1]*Department of Electrical and Computer Engineering, The University of Texas at Austin, 10100 Burnet Rd. Austin, TX, 78758, USA.*
[2]*Omega Optics Inc., 8500 Shoal Creek Blvd., Bldg. 4, Suite 200, Austin, TX, 78757, USA.*
[3]*yoo_eb@utexas.edu*
[4]*chenrt@austin.utexas.edu*

INDEX

Figure S1	The reconstructed signals from the standard SHFTS, which shows an aliasing error due to the bandwidth exceeded signal.	S-2
Description, Figure S2	Silicon-nitride strip waveguide design and simulation result.	S-2,3
Figure S3,4	Additional simulation results for sub-wavelength grating coupler (SWGC) design.	S-4,5
Description, Table S1, Figure S5	MMI splitter and combiner design and simulation result.	S-5,6
Description, Figure S6-8 Table S2	Si_3N_4 SHFTS device fabrication process and fabricated device images and layout.	S-6-8
Figure S9	The schematic and photograph of the broadband SuperK laser measurement setup.	S-8,9
Figure S10	The pictures of the SuperK laser coupling setup and the optical spectrum of the broadband SuperK laser coupled to the PM-SMF measured by OSA.	S-9
Figure S11	The output powers from the Si3N4 strip waveguides with various lengths for propagation loss measurement.	S-10
Figure S12	The coupling loss values as a function of coupling angle from 0 to 32 degree from the fabricated SWGC	S-10
Figure S13	Optical microscope images of light coupled SHFTS and MZI structures with background light on/off.	S-11
Figure S14	Output power measurement results using bandpass sampling SHFTS with different SWGC coupling conditions.	S-11,12
Figure S15	Broadband spectrum retrieval using bandpass sampling SHFTS by changing the grating coupling angles and polarizations.	S-12
Discussion	Further discussion about the on-chip broadband light source & detector integration and biosensing applications	S-13,14

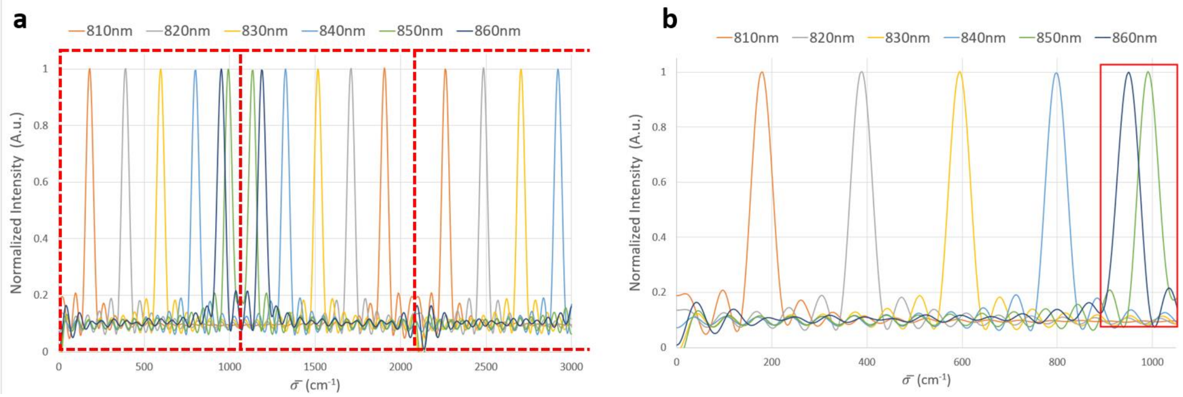

Figure S1. The reconstructed signals from the standard SHFTS. **a** The wavenumber-shifted copies of the original Fourier transform. **b** Zoomed-in spectrum within the bandwidth, which shows an aliasing error due to the bandwidth exceeded signal (λ_o =860 nm).

Silicon-nitride strip waveguide design and simulation

The Si_3N_4 strip waveguide comprises of the Si_3N_4 waveguide core and SiO_2 bottom cladding on Si substrate (Fig. S2a). To ensure the broadband fundamental transverse-electric (TE0) single mode operation covering the targeting wavelength range ($\lambda = 650\ nm$ to $1050\ nm$), the width and height of the Si_3N_4 waveguide core are optimized as w_{wg} = 500 nm and h_{core} = 220 nm by scanning the effective index of the waveguide as a function of the width as shown in Fig. S2g. Using the optimized design, the effective index of guiding TE0, TM0 and TE1 modes as a function of the wavelength are shown in Fig. S2e. The result shows that the TE0 single mode covers from λ =620 nm with the cut-off wavelength at 1030 nm. Also, to build the MZI structure with the minimum size of the footprint, the minimum radius of the 90° bend should be determined to ensure the low-loss across the whole wavelength range. By sweeping the bending radius from 1 μm, we designed the minimum bend radius of 20 μm to guide the fundamental TE mode up to λ =1030 nm as shown in Fig. S2f, indicating the same cut-off wavelength with the straight waveguide. Considering the propagation loss of the bent waveguide and the mode mismatching between the straight and bent waveguides, the total bending loss from 90◦ bend with r = 20 μm was calculated as 0.027 dB. The refractive index and electrical field (E-field) profiles of the optimized strip waveguide and bent waveguide are shown in Fig. S2b, c and d.

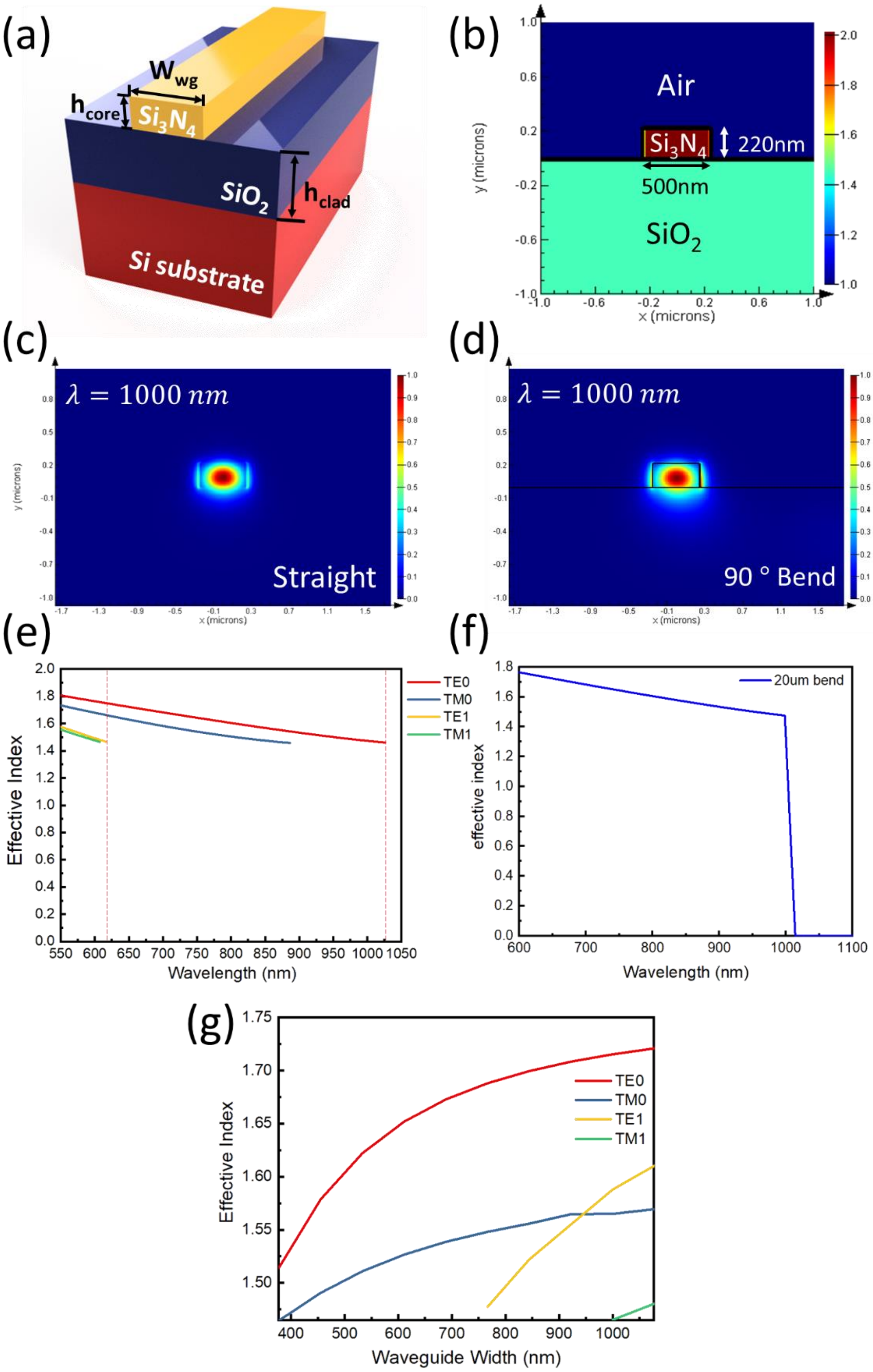

Figure S2. Si_3N_4 strip waveguide design and simulation results. **a** Schematic illustration of Si_3N_4 strip waveguide on SiO_2 bottom cladding with Si substrate. **b** Refractive index profile with w_{wg}=500 nm, h_{core}=220 nm, h_{clad}=2.8 μm. **c** E-field profiles of fundamental TE mode guiding through the straight waveguide and **d** 90-degree bent waveguide with r=20 μm at $\lambda = 1000\ nm$. **e** Effective index of guiding modes in optimized strip waveguide as a function of wavelength. **f** Effective index of 90-degree bent strip waveguide with r=20 μm. **g** Effective index of the Si_3N_4 strip waveguide as a function of w_{wg} at $\lambda = 800\ nm$.

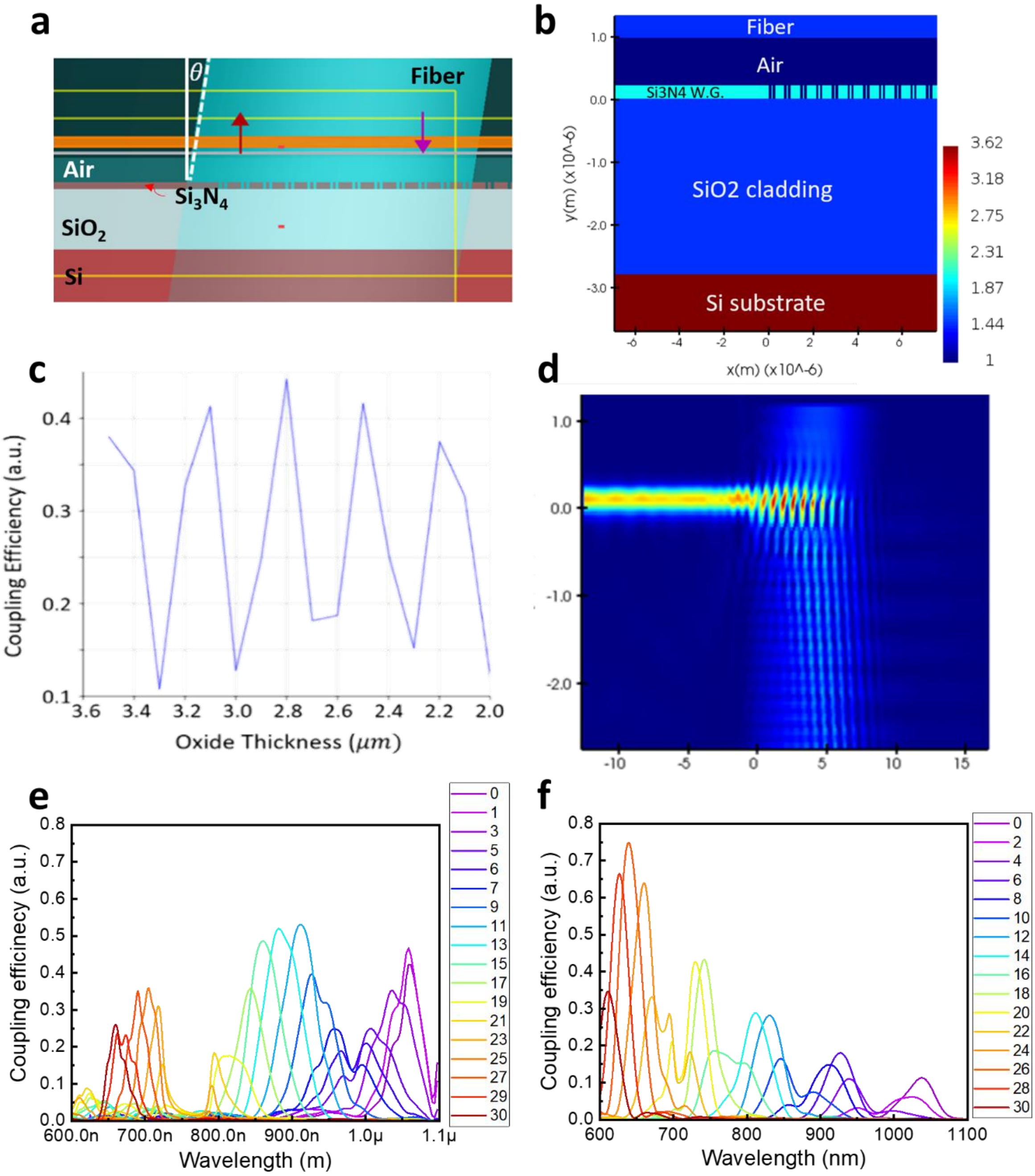

Figure S3. **a** FDTD simulation model including the SWGC structures and the fiber. **b** Refractive index profile of the optimized SWGC structure. **c** SWGC fundamental TE mode coupling efficiencies at $\lambda = 900\,nm$ as a function of the thickness of the SiO_2 bottom cladding. **d** E-field profile of the optimized SWGC for TE mode coupling. **e** Simulation results of SWGC TE mode coupling efficiencies with different coupling angles using fabricated dimension, and **f** TM mode coupling efficiencies.

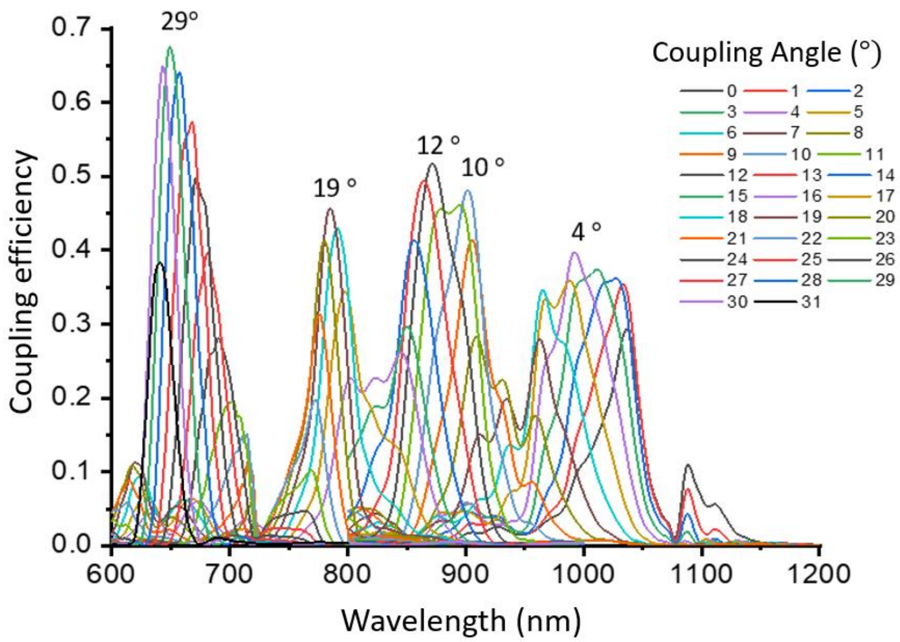

Figure S4. XY plot of the coupling efficiencies of the SWGC with different coupling angle from 0° to 31° as a function of wavelength.

MMI splitter and combiner design and simulation

Splitter and combiner are essential components to build MZI structure to equally divide and combine the light with 50:50 ratio. We optimized the MMI to minimize the loss using Lumerical Mode and FDTD simulations; the width of the splitter body is divided into 5 parts (w_{wg}, w_1, w_2, w_3, w_{gap}) and optimized the dimensions by the built-in particle-swarm algorithm to achieve the minimum loss for the fundamental TE mode across the wavelength range covering from 600nm to 1000 nm. The final optimized dimensions are shown in Table S1.

	w_{wg}	w_1	w_2	w_3	w_{gap}
**Width (nm)**	500	1128	1395	1360	200

Table S1. Optimized dimensions of MMI structure.

The schematic illustration of MMI structure and E-field simulation result with optimized dimensions are shown in Fig. S5a and b. To analyze the power-splitting performance, the S-parameters of output ports were calcultated (S21, S31) across $\lambda = 600 - 1000\ nm$. Fig. S5d shows the transmitted light intensites of output ports (port 2 and 3) with the normalized intensity of 1 from the input port (port 1); in other words, if there is no loss from the splitter, each output port should have 0.5 of transmitted power. The loss of the splitter is wavelength and polarization dependent, and we could get the minimum loss of ~0.5 dB at $\lambda = 800\ nm$, and it's getting increased to ~ 2 dB as the wavelength increases to 1000 nm as shown in Fig. S5d. Also, we checked the TM mode compatibility using the same design, and it turned out that the loss is about 3 dB higher than that of the TE mode operation as shown in Fig. S5e. To measure the loss values from the splitter experimentally, we cascaded 5 number of the MMI

splitters and measured the output powers from 32 output ports and calculated the loss values by the equation below.

$$Output\ power = \frac{Input\ power}{32} + Waveguide\ Propagation\ loss + 5 \times splitter\ loss \quad \text{(eq.1)}$$

The propagation loss from the waveguide was calculated based on the length of the guiding waveguide and the experimental propagation loss value, which was 0.335 cm and 2.4 dB/cm. As a result, the experimental splitter loss values were measured as ~0.6 dB, 1 dB and 2.2 dB at $\lambda_o = 750\ nm$, 905 nm and 1000 nm with TE mode, and 2.7 dB, 4 dB and 5.5 dB with TM mode, respectively. Furthermore, the same MMI structure can be used as a combiner in opposite direction. Fig. S5f shows the gradiant map of output power as functions of two input powers. Using the optimized structure, we could achieve ~1 dB combining loss when there are two equal inputs of the same phase at $\lambda = 800\ nm$.

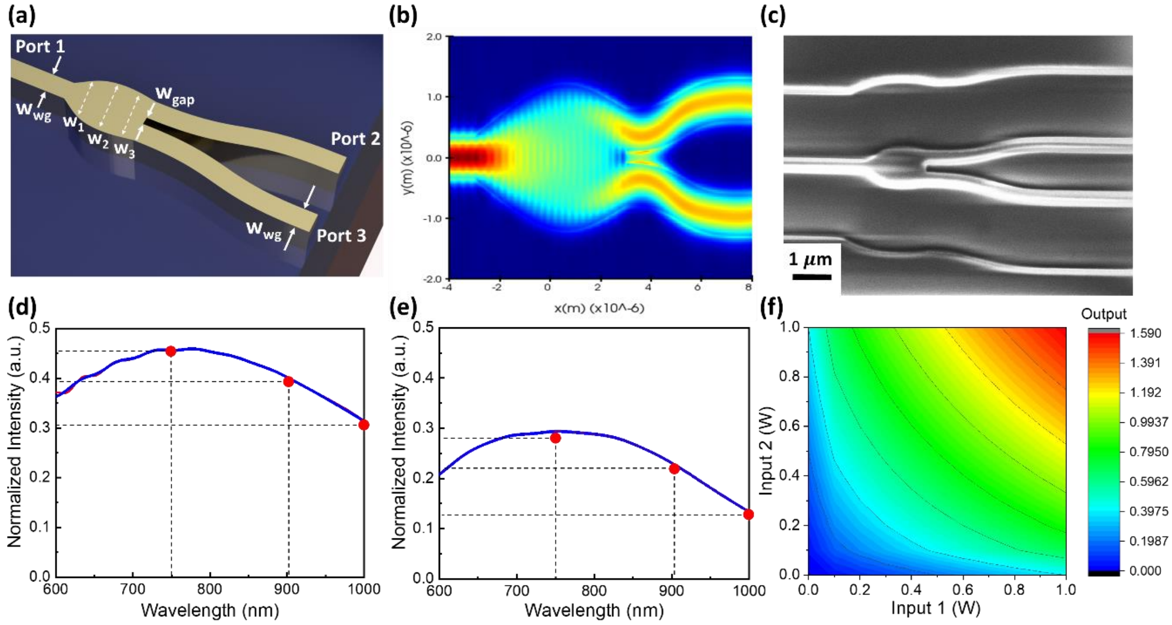

Figure S5. a 3D schematic illustration of the Si_3N_4 MMI design. **b** E-field of optimized MMI splitter simulation; Input port: port 1, Output ports: port 2 and 3. **c** SEM image of fabricated device. **d** TE mode and **e** TM mode simulation and measurement results of transmitted light intensities from port 1 to port 2 and 3 (S21 and S31) of the splitter; blue-line: simulation results, red-dot: measurement results. **f** Output power (port1) gradient map as a function of input powers (x-axis: port2, y-axis: port 3) of the combiner.

Si3N4 SHFTS device fabrication process

The Si_3N_4-on-SiO_2 wafers were prepared with a 220 nm thick LPCVD grown Si_3N_4 on a 2.8 μm thick SiO_2 bottom cladding on a silicon substrate (Fig. S6a) from Rogue Valley Microdevices Inc.. Then, ~400 nm thick Ebeam resist (ZEP-520A) is deposited on top of Si_3N_4 layer by spin-coating. The patterning is done by JEOL E-beam (JBX-6000FS) lithography tool, followed by developing in n-Amyl acetate for 2 min, and rinsing in isopropyl alcohol (IPA) (Fig. S6b). Following this, the pattern is transferred to Si_3N_4 layer by reactive-ion-etching (RIE) (Fig. S6c). Finally, the remaining resist and polymers are cleaned using removal PG and followed with cycles of Acetone/IPA post process treatment (Fig. S6d).

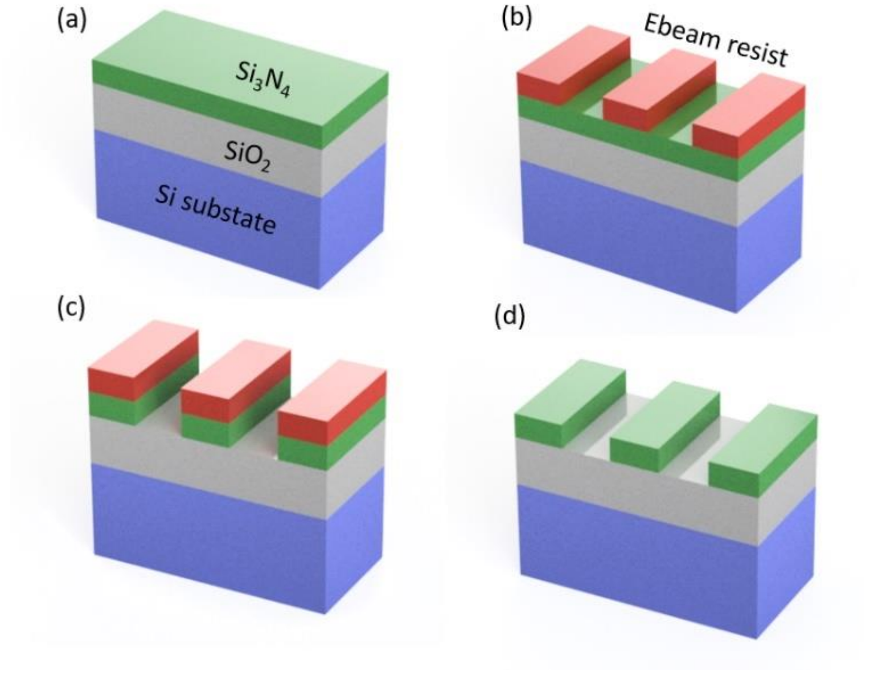

Figure S6. Schematic illustration of the fabrication process. **a** Si3N4-on-SiO2 wafer. **b** Deposit 400 nm E-beam resist (ZEP-520A) for e-beam lithography. **c** Etch Si3N4 layer with RIE. **d** Post-fabrication treatment with removal PG.

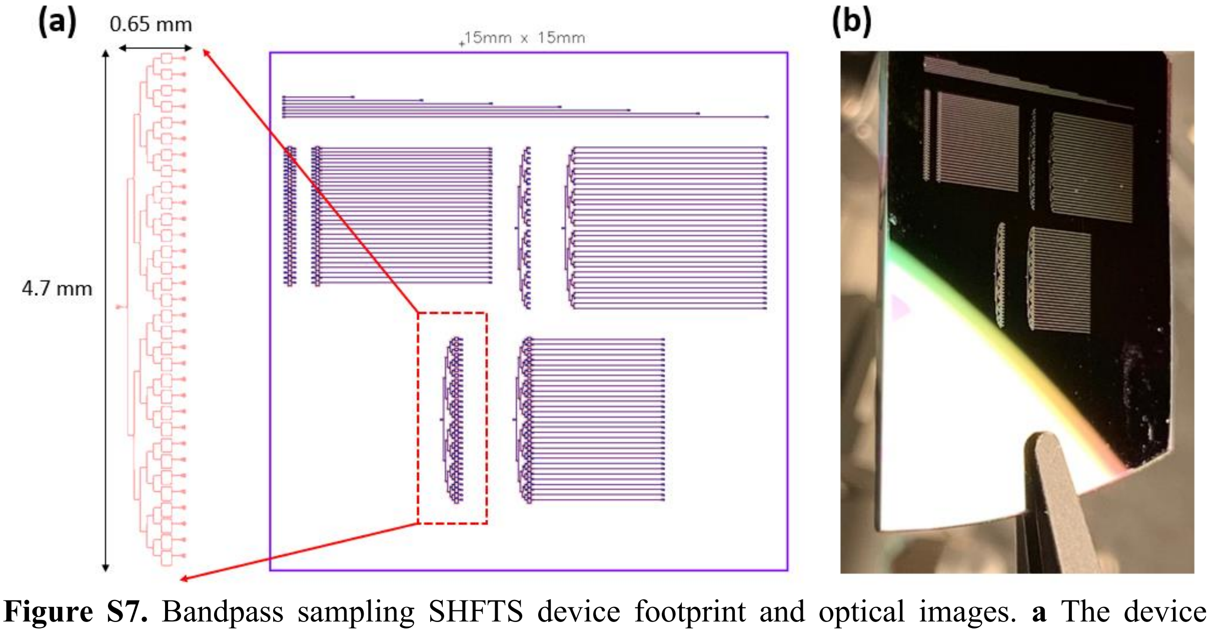

Figure S7. Bandpass sampling SHFTS device footprint and optical images. **a** The device footprint including characterization patterns. The size of SHFTS is around 4.7mm x 0.65 mm. **b** The picture of fabricated Si3N4 chip.

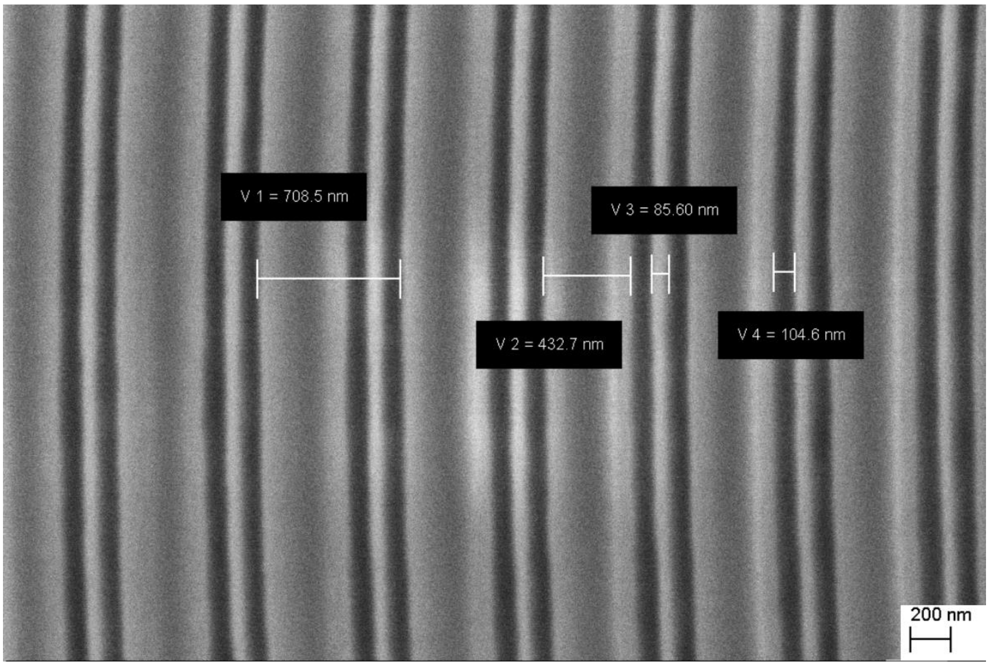

Figure S8. Zoomed-in SEM image of SWG structures in SWGC.

	Design	Fabrication	Deviation
**Si_3N_4 thickness (h_{core})**	220 nm	228.6 nm	+ 8.5 nm
**SiO_2 thickness (h_{clad})**	2.8 um	2.813 um	+ 13 nm
**Grating period (Λ)**	706 nm	708.5 nm	+ 2.5 nm
**Grating width (w_g)**	430 nm	433 nm	+ 3 nm
**SWG width (w_{swg})**	65 nm	85.6 nm	+ 25.6 nm

Table S2. SWGC dimension characterization.

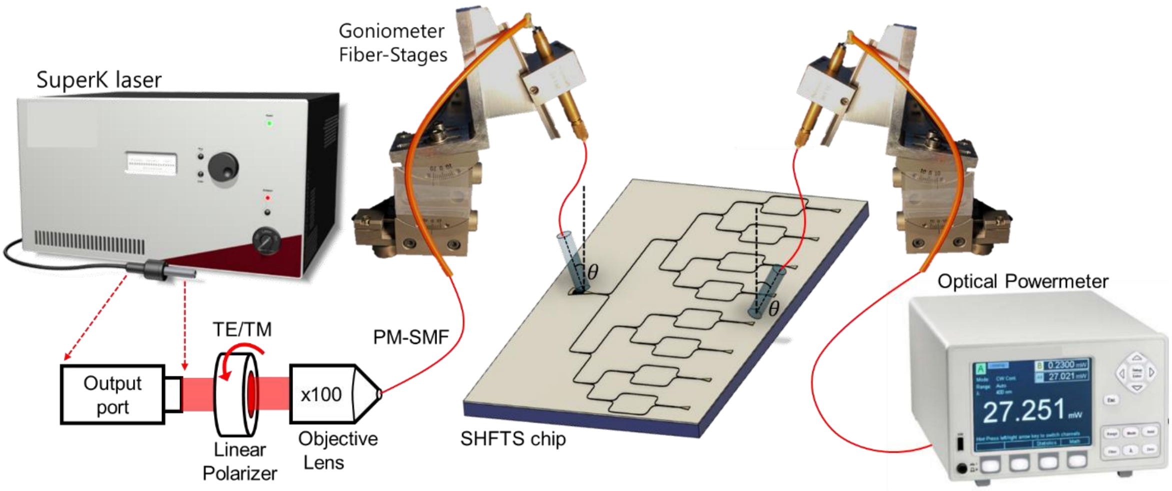

Figure S9. a A schematic diagram of the broadband SuperK laser measurement setup; PM-SMF: polarization-maintaining single mode fiber.

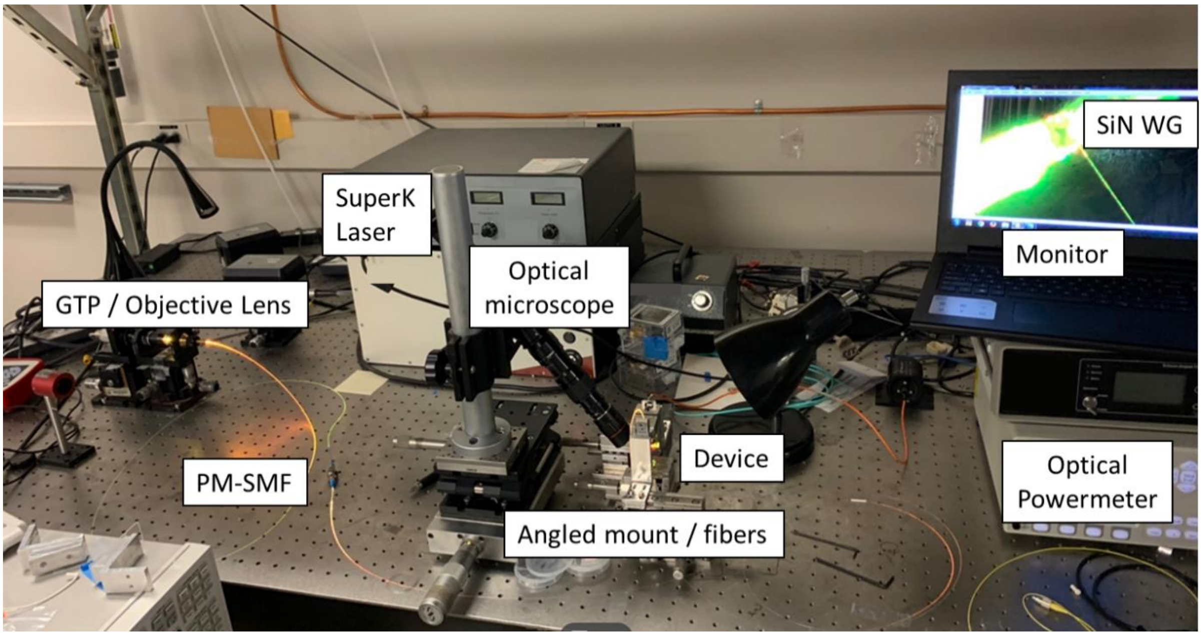

Figure S9. (cont.) b The picture of the broadband SuperK laser measurement setup; GTP: Glan-Thompson Linear Polarizer, PM-SMF: polarization-maintaining single mode fiber.

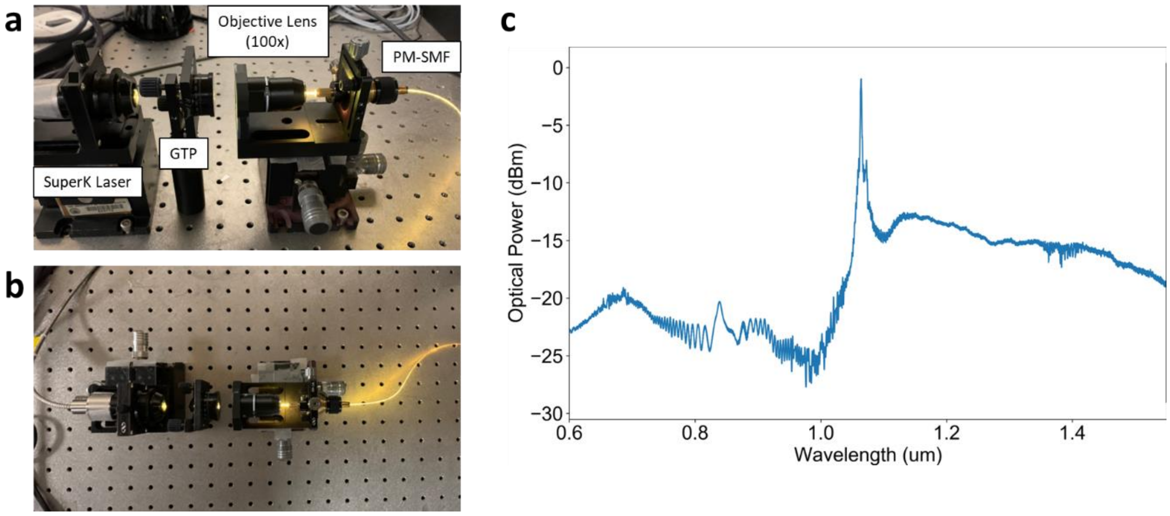

Figure S10. The pictures of the SuperK laser coupling setup with **a** side- and **b** top-view. **c** Optical spectrum of the broadband SuperK laser coupled to the PM-SMF measured by OSA; GTP: Glan-Thompson linear polarizer, PM-SMF: polarization-maintaining single mode fiber, OSA: optical spectrum analyzer.

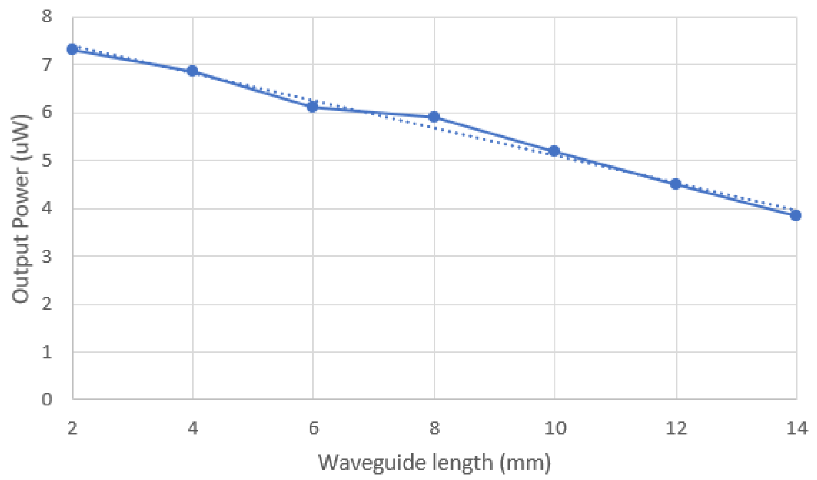

Figure S11. The output powers from the Si3N4 strip waveguides with various lengths for propagation loss measurement.

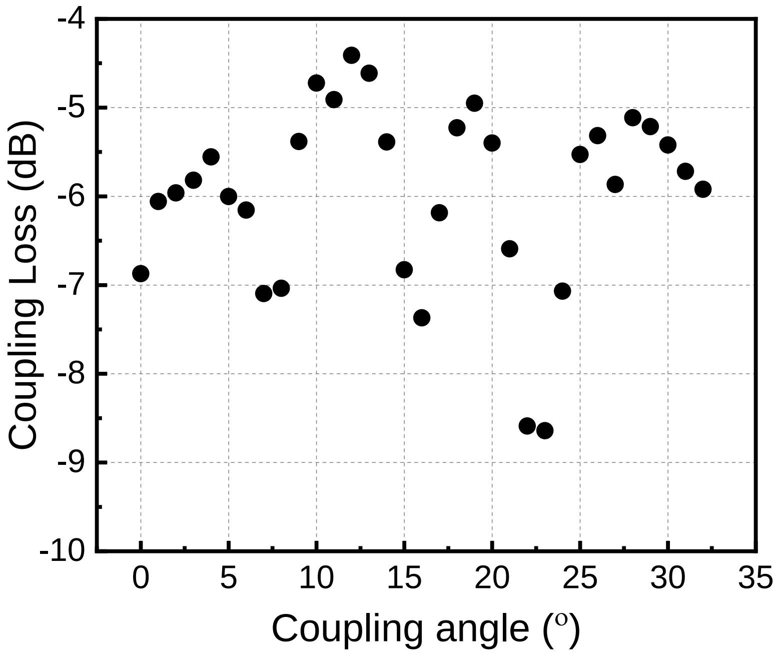

Figure S12. The measured coupling loss values as a function of coupling angle from 0 to 32 degree from the fabricated SWGC

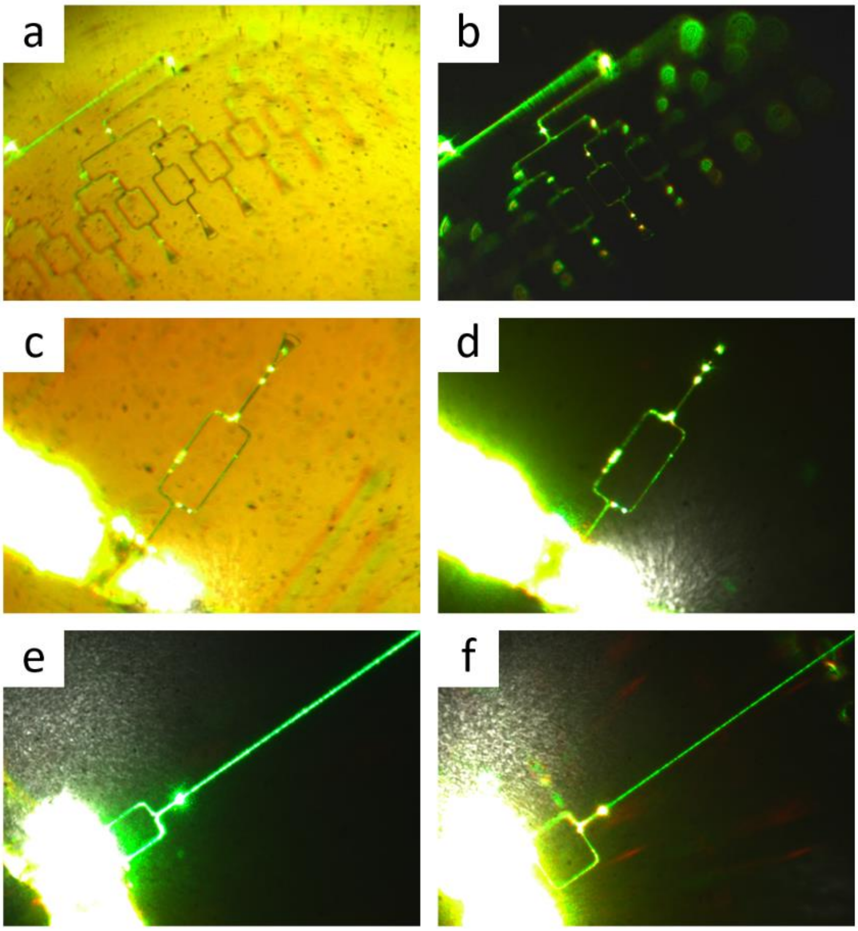

Figure S13. Optical microscope images of light coupled SHFTS and MZI structures with background light on/off. **(a-b)** SHFTS, **(c-f)** MZIs.

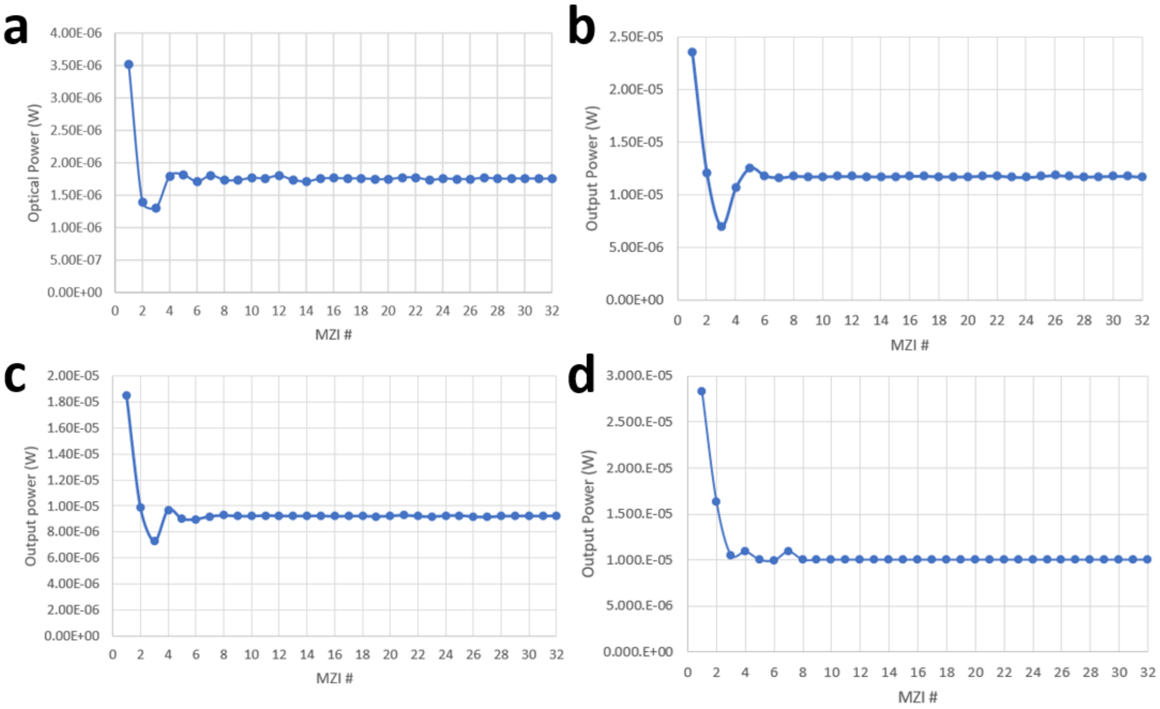

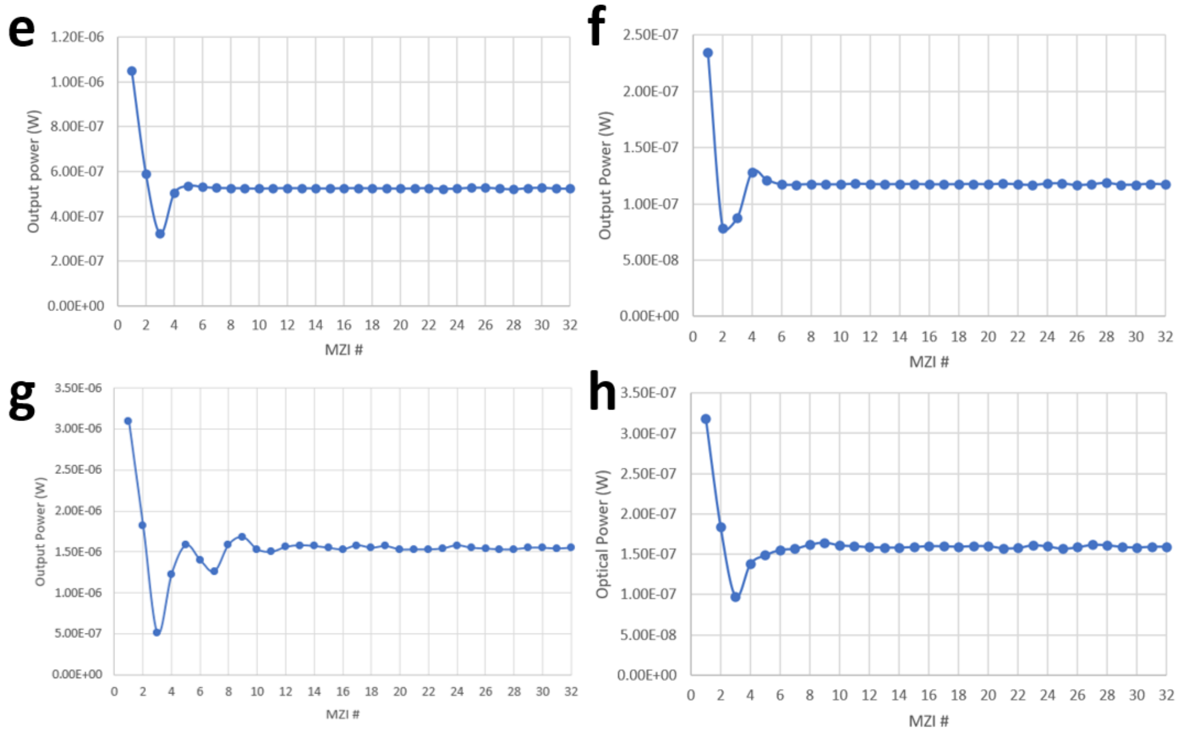

Figure S14. Output power measurement results using bandpass sampling SHFTS with different SWGC coupling conditions of **a** TE mode $\theta = 32°$, **b** TE mode $\theta = 25°$, **c** TM mode $\theta = 18°$, **d** TE mode $\theta = 20°$, **e** TM mode $\theta = 12°$, **f** TE mode $\theta = 14°$, **g** TE mode $\theta = 4°$, and **h** TE mode $\theta = 0°$.

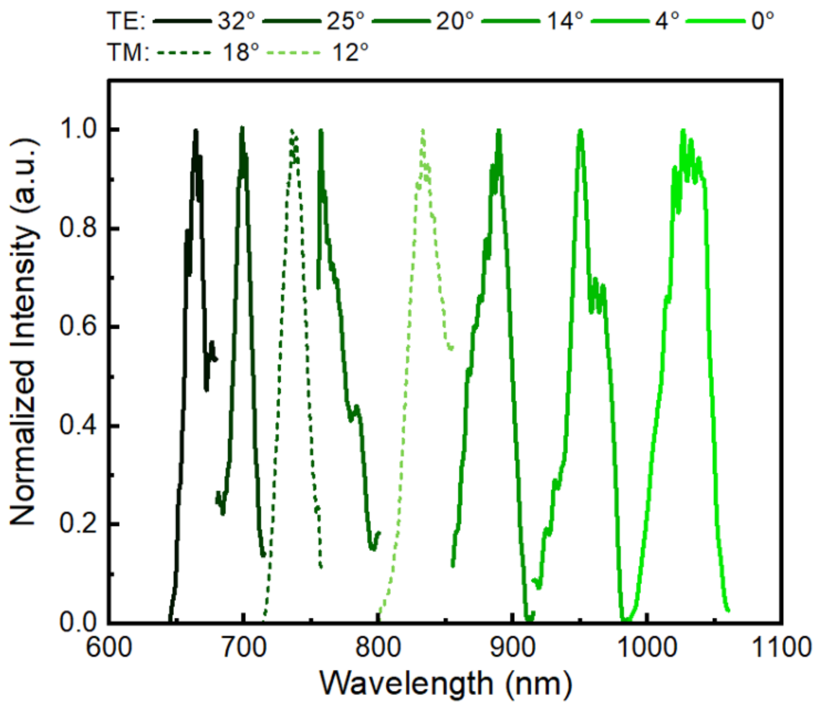

Figure S15. Broadband spectrum retrieval using bandpass sampling SHFTS by changing the grating coupling angles and polarizations (TE and TM).

Further Discussion:

On-chip broadband light source and photodetector integration

In the broadband spectrometer experiments presented in this work, we used the benchtop supercontinuum laser source with the objective lens to couple the light into the single-mode fiber as shown in Fig. S10. However, to realize the miniaturization of the whole FTS system, the integration of the on-chip broadband light source and photodetector arrays is required. Several broadband LED sources are available in the market, providing the spectral emission from $\lambda = 400\sim1000\,nm$; however, using the external LED sources is not an attractive solution to realize the on-chip spectrometer device ultimately, because of the high coupling losses and packaging challenges, and costs involved. Various researches have been conducted for the efficient PIC integrated light sources [1], including the heterogeneously integrated on-chip optically-pumped LED sources based on the InP platform [2], colloidal quantum dot integration on Si_3N_4 platform [3], on-chip supercontinuum sources [4], and else. Especially, Haolan et al. [4] reported the generation of an octave-spanning supercontinuum covering $\lambda = 488 - 978\,nm$ using a 1-cm-long Si_3N_4 waveguide with Ti:Sapphire pump laser at 795 nm. These advancements show a great promise for the monolithic integration of the broadband light source with the waveguide-based spectrometer device on the Si_3N_4 platform.

Moreover, to exploit the on-chip integrated broadband light source into the bandpass sampling SHFTS scheme, a proper method that can substitute the SWGC in this work should be devised as an on-chip tunable bandpass filter. Recent research reported the microelectromechanical (MEMS)-based tunable grating coupler [5], which is applicable in our SWGC design to actively control the coupling angle without moving the fiber or beam angle. Several other researches have reported the on-chip bandpass filter structures using the single-channel optical bandpass filter based on plasmonic nanocavities [6], gratings [20,21], and cascaded ring resonators [22] or MZI structures [23], which can be integrated with SHFTS configuration potentially. Hence, we expect a decent potential towards the PICs integrated bandpass sampling SHFTS with the on-chip broadband light sources. In addition, a lot of research have been presented the vertically- or end-coupled integrated photodetectors using various platforms such as germanium (Ge) on SOI [7], graphene [8], or other advanced 2D materials [9]. For the meaningful benchmarking, we focused on the CMOS-compatible photodetectors, especially for the arrayed waveguide integrated applications. Recently, Hongqiang et al. [7] reported the vertically coupled Ge PD array integrated with silicon waveguides for the on-chip arrayed waveguide grating (AWG) interrogator with around 90 % photon absorption and quantum efficiencies. The same concept can be used at the output ports of the MZI array in our SHFTS device to measure the output powers of each MZI interferogram, but the sensitivity and the noise level of the PD array should be explained experimentally to ensure the spectrometer performance. Based on these estimations, we expect further improvements to the compact, cost-effective, and reliable on-chip VIS-NIR spectrometer device based on the bandpass sampling SHFTS integrated with the on-chip broadband light source and PD array in a single chip, which enable the portable handheld spectrometer devices for the real-time in-vivo bio-photonic applications.

Towards compact-portable spectrometers for biosensing applications

The most essential value of a NIR tissue transparency window spectroscopy is that the light can penetrate into the skin beyond several millimeters of tissue benefit from the reduced tissue scattering and autofluorescence in these spectral ranges [10,11], therefore the optical

characteristics of various endogenous and exogenous tissue species can be measured based on the non-invasive assessment of the Raman scattering [12] or the absorption spectra to detect the glucose [13,14], tissue oxygen saturation level [15,16], and the skin-cancer detection [17], and various skin conditions [18]. However, the critical issues of the spectrometer system that require further elaboration for practical bio-sensing applications are the power and loss characterizations. Considering the entire device configuration for the in-vivo non-invasive biosensing applications which would be applied on top of the skin with a fiber-optic probe [17,19], a thorough investigation of the loss from the skin and device operation is required to assess how much input power should be utilized to reconstruct the signals. According to the recent study reported by Tianxing et al. [13], when linearly polarized NIR light illuminates the skin surface, approximately 58% (3.77 dB loss) of the light cannot interact with the tissues below the skin due to the absorption (53%) and direct reflection from the skin surface (5%), and the rest of the light penetrates the skin and may interact with tissues before and while being reflected back, but the majority of them (> 95%) will be depolarized. Since the SWGC in this work is polarization-dependent, the linear polarizer is required to filter TE or TM mode before the SWGC stage, which brings an additional ~3 dB loss. In total, there will be an inevitable ~7 dB loss from the skin approximately. Then, taking into account the experimentally measured maximum loss from the input SWGC (~8 dB), cascaded MMIs and waveguide propagation loss (~4 dB), we will have around 12 dB loss from the SHFTS device operation. Here, assuming the input source has a peak power of 10 dBm (10 mW), around -24 dBm (4 μW) can be transferred to each output photodetector in the array after being divided into 32 MZIs. However, we expect to have an additional loss from the skin to fiber-optic probe coupling, which must be further demonstrated experimentally. Taking all of this into account, we believe that this research constitutes the first step to realize fully integrated portable bio-spectrometer sensors.

References

1. A. Z. Subramanian, E. Ryckeboer, A. Dhakal, F. Peyskens, A. Malik, B. Kuyken, H. Zhao, S. Pathak, A. Ruocco, A. D. Groote, P. Wuytens, D. Martens, F. Leo, W. Xie, U. D. Dave, M. Muneeb, P. V. Dorpe, J. V. Campenhout, W. Bogaerts, P. Bienstman, N. L. Thomas, D. V. Thourhout, Z. Hens, G. Roelkens, and R. Baets, "Silicon and silicon nitride photonic circuits for spectroscopic sensing on-a-chip [Invited]," Photonics Res. **3** (5), B47-B59 (2015).
2. A. D. Groote, P. Cardile, A. Z. Subramanian, M. Tassaert, D. Delbeke, R. Baets, and G. Roelkens, "A waveguide coupled LED on SOI by heterogeneous integration of InP-based membranes", in *2015 IEEE 12th International Conference on Group IV Photonics (GFP)* (2015), pp. 31-32.
3. W. Xie, Y. Zhu, T. Aubert, Z. Hens, E. Brainis and D. V. Thourhout, "On-chip hybrid integration of silicon nitride microdisk with colloidal quantum dots," in *2015 IEEE 12th International Conference on Group IV Photonics (GFP)* (2015), pp. 159-160.
4. H. Zhao, B. Kuyken, S. Clemmen, F. Leo, A. Subramanian, A. Dhakal, P. Helin, S. Severi, E. Brainis, G. Roelkens, and R. Baets, "Visible-to-near-infrared octave spanning supercontinuum generation in a silicon nitride waveguide," Opt. Lett. **40** (10), 2177-2180 (2015).
5. W. Yu, S. Gao, Y. Lin, M. He, L. Liu, J. Xu, Y. Luo, and X. Cai, "MEMS-Based Tunable Grating Coupler," IEEE Photonics Technology Letters **31** (2), 161-164 (2019).
6. M. M. Najafabadi, S. Vahidi, H. Ghafoorifard, and M. Valizadeh, "Single-channel high-transmission optical band-pass filter based on plasmonic nanocavities," J. Opt. Soc. Am. B **37** (8), 2329-2337 (2020).
7. H. Li, S. Zhang, Z. Zhang, S. Zuo, S. Zhang, Y. Sun, D. Zhao, and Z. Zhang, "Silicon Waveguide Integrated with Germanium Photodetector for a Photonic-Integrated FBG Interrogator," Nanomaterials **10** (9), 1683 (2020).
8. J. Wang, Z. Cheng, Z. Chen, J.-B. Xu, H. K. Tsang, and C. Shu, "Graphene photodetector integrated on silicon nitride waveguide," Journal of Applied Physics **117** (14), 144504 (2015).
9. N. Flöry, P. Ma, Y. Salamin, A. Emboras, T. Taniguchi, K. Watanabe, J. Leuthold, and L. Novotny, "Waveguide-integrated van der Waals heterostructure photodetector at telecom wavelengths with high speed and high responsivity," Nature Nanotechnology **15** (2), 118-124 (2020).
10. R. R.-Kortum and E. S.-Muraca, "QUANTITATIVE OPTICAL SPECTROSCOPY FOR TISSUE DIAGNOSIS," Annual Review of Physical Chemistry **47** (1), 555-606 (1996).
11. S. Golovynskyi, I. Golovynska, L. I. Stepanova, O. I. Datsenko, L. Liu, J. Qu, and T. Y. Ohulchanskyy, "Optical windows for head tissues in near-infrared and short-wave infrared regions: Approaching transcranial light applications," Journal of Biophotonics **11** (12), e201800141 (2018).
12. J. Zhao, H. Lui, D. I. McLean, and H. Zeng, "Chapter 12 - Rapid Real-Time Raman Spectroscopy and Imaging-Guided Confocal Raman Spectroscopy for In Vivo Skin Evaluation and Diagnosis", in *Imaging in Dermatology*, edited by Michael R. Hamblin, Pinar Avci, and Gaurav K. Gupta (Academic Press, Boston, 2016), pp. 119-139.
13. T. Li, D. Bai, T. Prioleau, N. Bui, T. Vu, and X. Zhou, "Noninvasive glucose monitoring using polarized light", in *Proceedings of the 18th Conference on Embedded Networked Sensor Systems* (2020), pp. 544–557.
14. L. Tang, S. J. Chang, C.-J. Chen, and J.-T. Liu, "Non-Invasive Blood Glucose Monitoring Technology: A Review," Sensors-Basel **20** (23), 6925 (2020).

15. H. Y. Tsai, K. C. Huang, H. C. Chang, J. L. A. Yeh, and C. H. Chang, "A Noncontact Skin Oxygen-Saturation Imaging System for Measuring Human Tissue Oxygen Saturation," IEEE Transactions on Instrumentation and Measurement **63** (11), 2620-2631 (2014).
16. A. S. Kogler, T. V. Bilfinger, R. M. Galler, R. C. Mesquita, M. Cutrone, S. S. Schenkel, A. G. Yodh, and T. F. Floyd, "Fiber-optic Monitoring of Spinal Cord Hemodynamics in Experimental Aortic Occlusion," Anesthesiology **123** (6), 1362-1373 (2015).
17. M. Sharma, E. Marple, J. Reichenberg, and J. W. Tunnell, "Design and characterization of a novel multimodal fiber-optic probe and spectroscopy system for skin cancer applications," Review of Scientific Instruments **85** (8), 083101 (2014).
18. S. Prince and S. Malarvizhi, "Analysis of spectroscopic diffuse reflectance plots for different skin conditions," Spectroscopy **24**, 791473 (2010).
19. U. Utzinger and R. R.-Kortum, "Fiber optic probes for biomedical optical spectroscopy," Journal of Biomedical Optics **8** (1) (2003).
20. P. Sah and B. K. Das, "Photonic bandpass filter characteristics of multimode SOI waveguides integrated with submicron gratings," Appl. Opt. 57, 2277-2281 (2018).
21. B. Liu, Y. Zhang, Y. He, X. Jiang, J. Peng, C. Qiu, and Y. Su, "Silicon photonic bandpass filter based on apodized subwavelength grating with high suppression ratio and short coupling length," Opt. Express 25, 11359-11364 (2017)
22. K. Jinguji and M. Oguma, "Optical half-band filters," in Journal of Lightwave Technology, vol. 18, no. 2, pp. 252-259, Feb. 2000, doi: 10.1109/50.822800.
23. Xie, Yiwei, Geng, Zihan, Zhuang, Leimeng, Burla, Maurizio, Taddei, Caterina, Hoekman, Marcel, Leinse, Arne, Roeloffzen, Chris G.H., Boller, Klaus-J. and Lowery, Arthur J.. "Programmable optical processor chips: toward photonic RF filters with DSP-level flexibility and MHz-band selectivity" Nanophotonics, vol. 7, no. 2, 2018, pp. 421-454. https://doi.org/10.1515/nanoph-2017-0077
